# Continuous Pyruvate Supplementation Enhances Neuroprotective Resilience Against Kainate-Induced Status Epilepticus Through Metabolic Preconditioning

**DOI:** 10.3390/biom16060805

**Published:** 2026-05-29

**Authors:** Yong Jae Cho, Soo Jin Lee, Yuna Kim, Yeeun Kim, Seog-Young Kim, Kyunggon Kim, Dong-Cheol Woo, Hyun Ju Yoo, Joo-Yong Lee

**Affiliations:** 1 Asan Institute for Life Sciences, Asan Medical Center, Seoul 05505, Republic of Korea; us00887@amc.seoul.kr (Y.J.C.); soojin@mail.ulsan.ac.kr (S.J.L.); yuna00814@mail.ulsan.ac.kr (Y.K.); wbel56@mail.ulsan.ac.kr (Y.K.); sykim3@amc.seoul.kr (S.-Y.K.); kkkon1@amc.seoul.kr (K.K.); dcwoo@amc.seoul.kr (D.-C.W.); yoohyunju@amc.seoul.kr (H.J.Y.); 2 Department of Medical Science, University of Ulsan College of Medicine, Seoul 05505, Republic of Korea; 3 Department of Convergence Medicine, Asan Medical Center, Seoul 05505, Republic of Korea; 4 Department of Digital Medicine, University of Ulsan College of Medicine, Seoul 05505, Republic of Korea; 5 Department of Biochemistry and Molecular Biology, University of Ulsan College of Medicine, Seoul 05505, Republic of Korea

**Keywords:** epileptic seizures, epileptogenesis, neurochemical or energy metabolism, neurobehavioral comorbidity, prophylaxis, sodium pyruvate

## Abstract

Refractory status epilepticus refers to persistent and recurrent seizures unresponsive to medication, often leading to neuronal injury and neurobehavioral deficits. Studies have demonstrated that intraperitoneal bolus administration of pyruvate attenuates neuronal damage in rodent models of chemically induced status epilepticus (SE), while the precise neuroprotective mechanism remains to be further explored. This study investigated the neuroprotective effects of long-term supplementation with exogenous pyruvate against SE. When male C57BL/6 mice received 3% sodium pyruvate (SP) in the drinking water ad libitum for 20 weeks, they exhibited elevated levels of essential neurochemicals and energy metabolites in the brain compared to the control mice that received the equimolar saline solution. Following the intraperitoneal administration of kainate (KA) to induce severe SE, the SP-fed mice showed enhanced resistance to seizure activity, reduced neuronal injury, and improved neurobehavioral performance compared to the saline-fed mice. Regarding the molecular mechanisms underlying their neuroprotective properties, the levels of pyruvate metabolism-mediating proteins, neuronal and synaptic proteins, and neuroprotective proteins remained upregulated in the brains of the SP-fed mice following KA-induced SE. Conversely, the levels of pro-apoptotic and oxidative stress markers were suppressed. Collectively, this study indicates that long-term pyruvate supplementation may sustainably augment neurochemical and energy metabolism in the normal brain, thereby eliciting intrinsic neuroprotective properties. These effects contribute to preventing or ameliorating seizure activity, neuronal damage, and neurobehavioral deficits in mice following KA-induced SE, suggesting its prophylactic or therapeutic potential against epileptic seizures and SE through metabolic preconditioning.

## 1. Introduction

Epilepsy is a chronic brain disorder characterized by spontaneous, unprovoked seizures. Status epilepticus (SE) is a serious condition defined by seizures lasting longer than five minutes or recurrent seizures occurring in close succession without the patient regaining normal consciousness. While numerous seizures can be effectively controlled with antiseizure medications or surgery, approximately one-third of patients experience refractory SE. This condition does not respond to standard treatment and can cause permanent brain damage. SE also contributes to the development of temporal lobe epilepsy (TLE), the most common and severe form of acquired epilepsy [1]. In TLE, recurrent seizures result in hippocampal sclerosis, characterized by shrinkage and scarring, and widespread neuronal loss within the temporal lobe and surrounding limbic areas, including the hippocampus, amygdala, thalamus, and entorhinal cortex. Consequently, TLE can become increasingly resistant to antiseizure treatments, leading to persistently intractable conditions and permanent neurological damage. This progression is subsequently associated with additional comorbidities, such as behavioral, cognitive, and psychiatric impairments [2].

When kainate (KA), a potent excitotoxin that activates ionotropic α-amino-3-hydroxy-5-methyl-4-isoxazolepropionic acid (AMPA)/KA glutamate receptors, is administered intracerebrally or systemically, animals exhibit pathophysiological and behavioral phenotypes resembling the epileptic seizures observed in human TLE with SE [3,4]. The seizures are violent and persistent, leading to selective neuronal loss primarily within the limbic areas of the temporal lobes, specifically the hippocampal CA1 and CA3 pyramidal cell layers and the hilus [5,6]. Subsequently, hippocampal sclerosis develops, resulting in behavioral, cognitive, and motor impairments. Animal models of KA-induced SE have facilitated the investigation of diverse neuropathological features in brain tissue suffering from epileptic seizures, including metabolic disturbances, energy depletion, mitochondrial dysfunction, oxidative injury, neuroinflammatory damage, neuronal hyperexcitability, and metal toxicity. These factors can act individually or synergistically to contribute to epileptogenesis, encompassing the onset and progression of severe SE and TLE, ultimately resulting in neuronal injury. Therefore, KA-induced SE in rodents has served as an instrumental animal model for studying human SE and TLE, as well as epileptogenesis. It has been widely used in preclinical studies to investigate the neuropathophysiological processes of seizure episodes, along with the accompanying cognitive and behavioral impairments, and to further evaluate the efficacy of potential antiepileptogenic treatments [5,6].

Pyruvate (CH_3_COCOO^−^, the conjugate anion of pyruvic acid) has been consistently recognized as a promising neuroprotective agent since early biochemical and in vitro studies [7,8], which revealed its capacity to alleviate cellular oxidative damage, provide an effective alternative energy substrate, and modulate metabolic flux in response to cellular demands. Recent research has indicated that in vivo neuroprotective potential of pyruvate involves its ability to induce antioxidative or anti-inflammatory responses, improve mitochondrial energetics, and resist excitotoxicity or metal toxicity across a range of animal models of neuronal injury, including cerebral ischemia [9,10], traumatic contusions [11,12], and hypoglycemia [13]. In these studies, exogenous pyruvate, administered intraperitoneally as an acute large bolus of sodium pyruvate (SP), significantly reduced neuronal loss and the lesion volumes. Similarly, the neuroprotective potential of SP, administered as a single large bolus via intraperitoneal infusion, has been observed in rat models of epileptic seizures induced by KA [14] or pilocarpine [15,16]. However, the precise mechanisms by which exogenous pyruvate elicits neuroprotection in the brain during epileptic seizures or SE, and whether it possesses direct antiseizure potential, remain poorly understood.

Departing from previous studies that utilized an acute high-dose bolus intraperitoneal injection, this study initially investigated changes in the cerebral neurochemical and energy metabolites in normal mice following long-term ad libitum ingestion of pyruvate, which was achieved by supplementing the drinking water with SP over an extended period. Subsequently, the neuroprotective effects of this long-term, continuous ad libitum regimen were evaluated regarding seizure activity, neuronal injury and pathogenesis, and the associated neurobehavioral impairments in mice subjected to severe KA-induced SE.

## 2. Materials and Methods

### 2.1. Animal Studies and Ethics

Animals were maintained in accordance with the Guidelines for the Care and Use of Laboratory Animals of the Asan Institute for Life Sciences, Asan Medical Center (Seoul, Republic of Korea). Experimental protocols were reviewed and approved by the Institutional Animal Care and Use Committee (IACUC) of the Asan Institute for Life Sciences (approval number, 2022-12-248).

A total of 140 male C57BL/6 mice (5 weeks of age: Koatech, Pyeongtaek, Republic of Korea) were utilized across four independent rounds of experiments. Following acclimation for one week in specific pathogen-free facilities at the Asan Institute for Life Sciences, Asan Medical Center (Seoul, Republic of Korea), the animals were allocated to either a saline-fed control group or a pyruvate-fed experimental group based on body weight and housed in transparent plastic cages (W19 × L37 × H13 cm) with a density of four mice initially, then two or three animals depending on their growing body sizes, with ad libitum access to water and food at 22.0 ± 1.0 °C and a 12-h light/dark cycle.

### 2.2. Long-Term Continuous Ad Libitum Pyruvate Supplementation and Kainate-Induced Epileptic Seizures

Sodium pyruvate-supplemented drinking water (SP; 3% *w*/*v*, pH 7.1) was freshly prepared by dissolving sodium pyruvate (Sigma-Aldrich^®^, Merck, Darmstadt, Germany; Catalog number, P2256) in sterilized regular water, and replaced twice daily under light protection for the mice. As a control, the equimolar saline solution (pH 6.8) was prepared by adding 1.6% NaCl (Sigma-Aldrich^®^, Merck; S9625) to regular water. Consequently, mice consumed either 3% SP-supplemented drinking water (SP-fed; n = 62) or saline solution (saline-fed; n = 78) ad libitum for 20–22 weeks, starting from 6 weeks of age until euthanasia.

Subsequently, 25-week-old mice that had continued to ad libitum ingest either 3% SP-supplemented water (SP-fed/KA-injected) or saline (saline-fed/KA-injected) for the preceding 20 weeks were injected with kainate (KA; 40 mg/kg body weight in normal saline, pH 7.0; Tocris Bioscience, Minneapolis, MN, USA; #7065) into the peritoneal cavity (i.p.) to induce severe and sustained seizures without the administration of an anticonvulsant [17,18]. For the sham control, the mice maintained on either 3% SP-supplemented water (SP-fed sham) or saline (saline-fed sham) received the equivalent volume of normal saline instead of KA.

Seizure development and severity were assessed using behavioral stage classification criteria, with some modifications [17,18], as follows: stage 0, normal behaviors; stage 1, staring with immobility or hypo-responsiveness; stage 2, abrupt, impatient responses, digging, head nodding, twitching, tail extension, stretching, or myoclonic jerks, or rigid posture; stage 3, forelimb clonus, rearing and falling, or loss of posture and body balance; stage 4, recurrent seizures, excessive hyper-responsiveness, or violent behaviors such as jumping, circling, and rolling; and stage 5, death.

### 2.3. Postmortem Brain Evaluations

Mice were euthanized by cervical translocation 48 h after KA injection, and cerebral tissues were collected, snap-frozen in liquid nitrogen, and stored at −80 °C for further studies. Coronal brain slices (12 μm thick) were obtained using a cryostat (Leica Biosystems, Wetzlar, Germany) and mounted on poly-L-lysine-coated glass slides (Paul Marienfeld, Lauda-Königshofen, Germany), which were subsequently cryopreserved at −80 °C.

Brain slices were stained with 0.1% acid fuchsin (Sigma-Aldrich^®^, Merck; F8129) and 0.5% cresyl violet (Sigma-Aldrich^®^, Merck; C5042) to detect acidophilic and pyknotic dead neurons, respectively, and examined and photographed using a light microscope (ECLIPSE 80i; Nikon, Tokyo, Japan) equipped with a DS-Fi1/DS-U2 digital camera and NIS-Elements F2.20 image-analysis software (Nikon). To assess neuronal death, acidophilic (acid fuchsin-stained) neurons were counted in the cortical and hippocampal CA1 and CA3 regions of both hemispheres within five tissue slices taken every 200 μm, beginning at bregma −1.3 mm.

To visualize zinc accumulation in degenerating or dead neurons, slices were stained with the zinc-specific fluorescent probe TFLZn [N-(6-methoxy-8-quinolyl)-p-carboxybenzoylsulfonamide; Sigma-Aldrich^®^, Merck; T3570]. Without fixation, the slices were incubated with TFLZn (0.1 mM) in Tris buffer (pH 8.0) in the dark for 90 s. Following brief washing with normal saline, the stained slices were examined and photographed using a fluorescence microscope (ECLIPSE 80i) with a UV-2A filter (dichroic mirror: 400 nm; excitation filter: 355/50 nm; barrier filter: 410 nm).

### 2.4. Immunofluorescent Staining for Neuronal Nuclei (NeuN) Protein

To examine the expressive appearance of neuronal nuclei protein (NeuN, a.k.a. Fox-3), the brain tissue slices were initially fixed in 4% paraformaldehyde (PFA) in phosphate-buffered saline (PBS, pH 7.2), blocked with blocking buffer (3% normal serum and 0.3% Triton X-100 in PBS), and incubated with the anti-NeuN antibody (Chemicon^®^, Merck; MAB377; 1:250). Thereafter, the slices were immunofluorescently stained using Alexa Fluor 542/555-conjugated secondary antibodies (Invitrogen™, Thermo Fisher Scientific, Waltham, MA, USA), and examined and photographed under a fluorescence microscope (Eclipse 80i) equipped with a TRITC filter (dichroic mirror, 565 nm; excitation filter, 540/25 nm; barrier filter, 605/55 nm).

### 2.5. Immunoblotting Analysis

The primary antibodies used for immunoblotting were as follows: anti-3-nitrotyrosine (3-NT: Invitrogen™, Thermo Fisher Scientific; A21285; dilution, 1:1000), anti-4-hydroxynonenal (4-HNE; Bioss, Woburn, MA, USA; bs-6313R; 1:1000), anti-β-actin (Santa Cruz Biotechnology, Dallas, TX, USA; sc-47778; 1:1000), anti-β-tubulin (Santa Cruz Biotechnology; sc-101527; 1:1000), anti-caspase-3 (Cell Signaling Technology, Danvers, MA, USA; #9662; 1:1000), anti-excitatory amino acid carrier 1 (EAAC1, a.k.a. excitatory amino acid transporter 3, EAAT3: Bioss; bs-1752R; 1:1000), anti-glucose transporter 3 (GLUT3, a.k.a. SLC2A3: Invitrogen™, Thermo Fisher Scientific; PA5-99486; 1:1000), anti-glutathione peroxidase-4 (GPX4: Invitrogen™, Thermo Fisher Scientific; PA5-102521; 1:1000), anti-heat shock protein 70 (HSP70; Enzo Biochem, Farmingdale, NY, USA; ADI-SPA-810; ADI-SPA-810; 1:1000), anti-inducible nitric oxide synthase (iNOS, a.k.a. NOS2: Invitrogen™, Thermo Fisher Scientific; PA1-036; 1:1000), anti-MCT1 (a.k.a. SLC16A1: Invitrogen™, Thermo Fisher Scientific; PA5-76687; 1:1000), anti-monocarboxylate transporter 2 (MCT2, a.k.a. SLC16A7; PA5-77498; 1:500), anti-MCT4 (a.k.a. SLC16A3: Invitrogen™, Thermo Fisher Scientific; PA5-106683; 1:500), anti-mitochondrial pyruvate carrier 1 (MPC1, a.k.a. brain protein 44-like protein, BRP44L: Invitrogen™, Thermo Fisher Scientific; PA5-116938; 1:1000), anti-MPC2 (a.k.a. brain protein 44, BRP44: Invitrogen™, Thermo Fisher Scientific; PA5-116939; 1:1000), anti-NeuN (Chemicon^®^, Merck; MAB377; 1:1000), anti-p47phox (a.k.a. neutrophil cytosol factor 1, NCF1: Cell Signaling Technology; #63290; 1:1000), anti-p67phox (a.k.a. neutrophil cytosolic factor 2, NCF2: Cell Signaling Technology; #95781; 1:1000), anti-poly(ADP-ribose) polymerase-1 (PARP-1: Cell Signaling Technology; #9542; 1:500), anti-postsynaptic density protein 95 (PSD95: Invitrogen™, Thermo Fisher Scientific; #51-6900; 1:1000), anti-pyruvate dehydrogenase (PDH: Cell Signaling Technology; #3205; 1:500), anti-sirtuin 1 (Sirt1: Sigma-Aldrich^®^, Merck; #04-1557; 1:500), anti-superoxide dismutase-1 (SOD-1, a.k.a. Cu/Zn superoxide dismutase, Cu/Zn SOD: Invitrogen™, Thermo Fisher Scientific; MA1-105; 1:1000), and anti-synaptophysin (SYP: Sigma-Aldrich^®^, Merck; MAB329; 1:1000).

For immunoblotting analysis, mouse cerebral tissues containing the cortex and hippocampus were homogenized in PRO-PREP™ protein extraction solution supplemented with protease inhibitors (1 mM phenylmethylsulfonyl fluoride, 1 mM ethylenediaminetetraacetic acid, 1 mM pepstatin A, 1 mM leupeptin, and 1 mM aprotinin; iNtRON Biotechnology, Seongnam, Republic of Korea; #17081) and centrifuged to collect the proteins. After boiling in the sample buffer [62.5 mM Tris-HCl, pH 6.8, 2% sodium dodecyl sulfate (SDS), 10% glycerol, 0.01% bromophenol blue, 5% mercaptoethanol, dithiothreitol (DTT)], proteins were separated by 10–15% SDS–polyacrylamide gel electrophoresis (SDS-PAGE: Bio-Rad Laboratories, Hercules, CA, USA), transferred to polyvinylidene fluoride (PVDF) membranes (Amersham Hybond^®^, Cytiva, Marlborough, MA, USA), and blocked with 5% skim milk (Bio-Rad Laboratories) and 1% bovine serum albumin (BSA; Bovogen, Melbourne, Australia) in TBS-T buffer (10 mM Tris-HCl, pH 7.4, 150 mM NaCl, 0.1% Tween 20). Immunological detection was performed using a primary antibody, followed by incubation with a horseradish peroxidase (HRP)-conjugated secondary antibody (Santa Cruz Biotechnology). Proteins were detected using Immobilon Western Chemiluminescent HRP Substrate (Millipore^®^, Merck; WBKLS) and a Chemiluminescence Imaging System (Davinch-K, Seoul, Republic of Korea). Protein intensities were assessed using ImageJ software (Version 1.54p; National Institutes of Health, Bethesda, MD, USA), and the signals were normalized to those of β-actin or β-tubulin on the same blots.

### 2.6. In Vivo Proton Magnetic Resonance Spectroscopy (^1^H-MRS) for Measurement of Metabolic Neurochemicals in the Brain

Following the 20-week period of SP or saline supplementation, the mice were scanned alive using magnetic resonance imaging and spectroscopy (MRI/MRS) while lying prone on a custom-made cradle with the head firmly secured by a palate holder equipped with an adjustable nosecone and ear bars, all of which were made of high-density polyethylene, under 2.5% isoflurane anesthesia. Each scan took less than 60 min. The scans used a horizontal 9.4 T/160 mm MR scanner (Agilent Technologies, Palo Alto, CA, USA) equipped with a 400 mT/m gradient system and a two-channel mouse brain coil. Axial and coronal T2-weighted MR images were acquired using fast spin echo (FSE) sequences with the following parameters: a repetition time (TR) of 4000 ms, an echo time (TE) of 60 ms, a data matrix of 256 × 256, slice thicknesses of 0.8 mm (axial) and 0.6 mm (coronal), two excitations (NEX), and 20 axial and 10 coronal slices. The volume of interest (VOI) was adjusted to target the left hippocampal region using point resolved spectroscopy (PRESS) as follows: TR of 5000 ms, TE of 13.4 ms, an average of 384 complex data points of 2048, a spectral width of 5000 Hz, and a VOI size of 1.2 × 1.5 × 2.0 mm^3^ (3.6 μL). Following VOI positioning, automated shimming, water signal suppression, and transmit-receive gain were all optimized. Water signal suppression was achieved using the variable power and optimized relaxation delays (VAPOR) method. Water-unsuppressed spectra were used to determine metabolite concentrations as an internal reference.

In vivo proton MR spectra were analyzed using the Linear Combination Model software (LCModel, version 6.3-1D; Stephen Provencher, Oakville, ONT, Canada) [19], which incorporated a simulated basis set including spectra for the following metabolic neurochemicals: alanine (Ala), aspartate (Asp), creatine (Cr), phosphocreatine (PCr), γ-aminobutyric acid (GABA), glucose (GLU), glutamine (Gln), glutamate (Glu), glycerophosphocholine (GPC), phosphorylcholine (PCh), glutathione (GSH), myo-inositol (MI), lactate (Lac), N-acetylaspartate (NAA), N-acetylaspartylglutamate (NAAG), scyllo-inositol (scyllo-Ins), taurine (TRN), total choline (tCho: GPC + PCh), macromolecules, and lipids. Analyses were performed in the frequency domain between 0.2 and 4.3 ppm using water-suppressed raw data (free induction decays, FIDs). The concentrations of metabolic neurochemicals were calculated using the unsuppressed water signal as a concentration reference and were considered reliable when the Cramér-Rao lower bounds (CRLBs) exhibited a percent standard deviation (%SD) of less than 20% [20]. Consequently, absolute concentrations (mM) were determined for PCr, GABA, Gln, Glu, GSH, MI, NAA, TRN, tCho, total NAA (tNAA: NAA + NAAG), total creatine (tCr: PCr + Cr), and Glu and Gln complex (Glx).

### 2.7. Targeted Metabolomic Analysis of Energy Metabolism Using Liquid Chromatography–Tandem Mass Spectrometry (LC-MS/MS)

Metabolites involved in cerebral energy metabolism [glycolysis, gluconeogenesis, tricarboxylic acid (TCA) cycle, oxidative phosphorylation (OXPHOS), and pentose phosphate pathway (PPP)] were analyzed using LC-MS/MS-assisted targeted metabolomics.

The mice were killed the following day after the in vivo MRI/MRS scanning, and their brains were rapidly removed. The brain tissues (200 mg), including the cortex and hippocampus, were immediately collected and homogenized using a TissueLyzer (Qiagen, Hilden, Germany) in four volumes of cold chloroform:methanol (2:1, *v*/*v*) solution to which 100 μL of 10 μM Glutamine-^13^C_5_ (Sigma-Aldrich^®^, Merck; #605166) was added as an internal standard (IS). The samples were centrifuged to collect the supernatant, which was dissolved in water and centrifuged again. The upper fractions containing polar metabolites were dried under vacuum and stored at −20 °C. The lyophilized metabolite samples were reconstituted with H_2_O:acetonitrile (50:50, *v*/*v*) solution before LC-MS/MS analysis using a 1290 Infinity II ultrahigh-performance liquid chromatograph (HPLC; Agilent Technologies, Santa Clara, CA, USA) coupled with a Qtrap 5500 (AB Sciex, Framingham, MA, USA) and a reverse-phase column (Synergi Fusion RP 50 × 2 mm; Phenomenex, Torrance, CA, USA). Mobile phases A and B comprised 5 mM ammonium acetate in H_2_O and 5 mM ammonium acetate in acetonitrile, respectively. The elution gradient was as follows: hold at 0% B for 5 min, increase from 0% to 90% B over 2 min, hold at 90% B for 8 min, decrease from 90% to 0% B over 1 min, and finally, hold at 0% B for 9 min. The LC flow rate was set at 70 μL/min, except for 7–15 min when it was increased to 140 μL/min, whereas the column temperature was maintained at 23 °C. Multiple reaction monitoring (MRM) was conducted in negative ion mode, and the extracted ion chromatogram (EIC) was used for quantitative evaluation. The peak area of each metabolite under the EIC curve was normalized to the IS peak area, and the relative ratio (fold-times) was represented as the corrected metabolite abundance.

### 2.8. Assessment of Neurobehavioral Performance

Following the subsidence of recurrent seizures three days after the onset of KA-induced SE, neurobehavioral evaluations were undertaken using separate animal cohorts for each motor and cognitive test. All test procedures were performed blindly under consistent experimental and environmental conditions, adhering to the same skilled experimenter-same operation principle. Animals were allowed continuous ad libitum access to either 3% SP-supplemented drinking water or saline throughout the neurobehavioral testing period.

Muscle strength and neuromuscular performance were assessed using a grip strength meter (Panlab, Barcelona, Spain). The mice were placed on a metal mesh grid connected to the meter and allowed to grab it with all four paws while gently pulling back horizontally with their tails. The peak force applied to the grid when all four paws released their grip was recorded as the maximum grip strength [in gram-force (gf)] of the animal. The test was repeated three times with a 5-min rest interval between trials. The mean value from the three trials was used to determine the grip strength of each animal, which was then normalized to its body weight (in gf/g).

Mice were additionally evaluated for motor balance, coordination, and motor learning to use the balance beam and rotarod tests.

The balance beam was a flat wooden bar (W2 × L80 cm) supported horizontally between two columns that were 40 cm high, and an escape chamber was placed at one end of the bar. Thirty minutes before performing the first test trial, the mice were allowed to freely explore the balance beam apparatus for 90 s. The mice were then encouraged to run from the starting end to the escape chamber at the other end while being subjected to sudden intense illumination and roar at the starting point. When a mouse fell off the beam, another trial was performed. If a mouse lingered on the beam but did not reach the chamber, a cutoff time of 90 s was imposed. The trial was performed three times with a 10-min resting interval between each test. The time taken by each mouse to traverse the beam and enter the escape chamber was recorded.

For the rotarod test, the animals were first trained to walk for 3 min on a cylindrical rod (30 mm in diameter) of the rotarod (Ugo Basile, Gemonio, Italy) that was set to rotate in ramp mode (with an acceleration time of 60 s and a maximum speed of 20 rpm), and then received an additional 3 min of walking training at a constant speed of 15 rpm. Fifteen minutes after these training sessions, the mice were tested to walk on the rod for a maximum of 200 s while it rotated at a constant speed of 15 rpm. The time spent by the mice walking on the rotating rod without falling was recorded. The test was repeated three times daily, with a 10-min break between each test, for three consecutive days. The mean value of the three trials was calculated to determine the time latency of each animal.

The spatial working memory of the animals was evaluated using the spontaneous alternation T-maze test [21], which comprised a T-shaped opaque plastic apparatus with a central start arm (W7 × L30 × H15 cm), both side goal arms (W7 × L30 × H15 cm), an in-between connecting square (W7 × L7 × H15 cm), and guillotine doors set in the middle of the start arm and on the entries of both goal arms. For the trial, after placement in the opposite direction at the distal end of the start arm, the mice could freely explore the T-maze and spontaneously choose a goal arm for entry. The door on the start arm was quietly closed to prevent the animals from returning when they entered the connecting square to choose a goal arm to explore, and the door on the goal arm was closed immediately after their four paws and tails completely passed over the entry line of the goal arm. The mice explored or stayed there ad libitum for 30 s, followed by six trials in a row. Each trial had a 3-min limit; unresponsive animals were not scored. The choice to enter either the opposite or the same arm as in the previous trial was used to measure the percentage of correct or incorrect alternation, which reflected working memory performance. In addition, the propensity to choose the same sidearm over trial repetitions was assessed to evaluate ipsilateral preference, which may have been influenced by KA-induced unilateral brain lesions.

Mice were also tested for spatial learning and memory performance in the Morris water maze (MWM), comprising a circular plastic pool (120 cm in diameter) with three visual cues on the wall and a cylindrical escape platform (10 cm in diameter) in the northwest (NW) quadrant, 0.5 cm below the surface of opaque water maintained at 21.0 ± 10 °C. The animals were trained for seven days to swim and locate a hidden escape platform in the pool. Three trials were conducted daily, each beginning in the northeast (NE), southeast (SE), or southwest (SW) quadrant and lasting for 60 s. The mean time taken to reach the escape platform in the daily triplicate trials was determined as the escape latency of each tested animal. Three hours after the final performance, the mice underwent a free swim probe test for 60 s, starting from the SE quadrant, with the escape platform removed. During this trial, the time spent swimming, path taken, distance traveled in the pool, and frequency with which the target zone was crossed to locate the removed platform were monitored using the SMART Video Tracking System (Harvard Apparatus, Holliston, MA, USA).

### 2.9. Statistical Analysis

Data were presented as the mean ± standard deviation (SD) or the mean ± standard error of the mean (SEM). Differences between groups were determined using an analysis of variance (ANOVA) with a post hoc test or a *t*-test by means of Prism software (GraphPad Software, Boston, MA, USA; version, 8.0). Statistical significance was defined at *p* < 0.05.

## 3. Results

### 3.1. Safety and Tolerance of Long-Term Ad Libitum Administration of Exogenous Pyruvate via Drinking Water

To determine a safe and effective ad libitum dosage for long-term administration, the effects of SP supplementation in drinking water at concentrations of 3% (*w*/*v*; pH 7.1), 5% (pH 6.8), and 9% (pH 6.7) (n = 5 per group), which had been arbitrarily selected, were preliminarily evaluated over a three-month period. The 9% SP concentration proved fatal, resulting in severe growth retardation, steep body weight loss, and behavioral impairments, with all tested subjects dying within one month. In contrast, both the 3% and the 5% SP concentrations were well tolerated and safe for the preliminary experimental period; however, the 3% concentration was ultimately selected for the long-term trial regimen to minimize the risk of adverse effects during prolonged administration.

**Figure 1:**
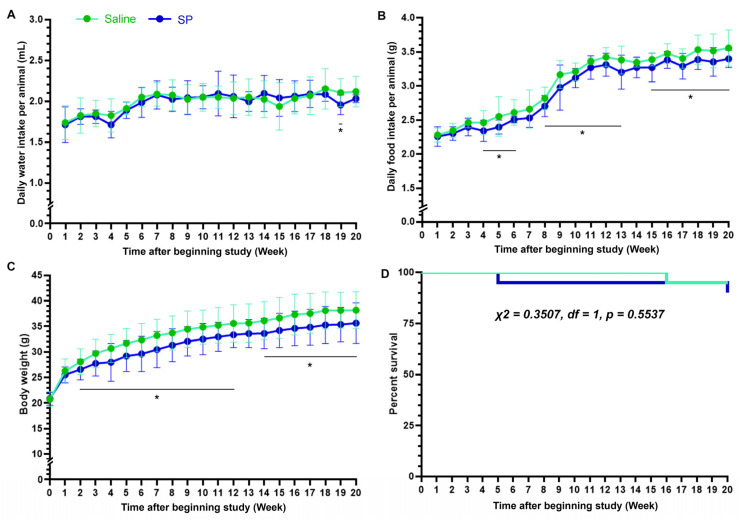
Effects of long-term drinking of sodium pyruvate (SP)-supplemented water on life and physical parameters. Daily water (**A**) and food (**B**) intake, body weight (**C**), and survival (**D**) were measured weekly in normal male C57BL/6 mice that continued to drink either 3% SP-supplemented water (SP-fed; blue) or 1.6% saline solution (saline-fed; green) ad libitum for 20 weeks. The study began with 40 mice evenly divided into two groups (therefore, n = 20 for each group). The number of animals used for weekly statistical comparison was subsequently revised for mortality as one death occurred among the saline-fed mice at week 16 (n = 19 thereafter) and two deaths among the SP-fed mice at weeks 5 (n = 19 thereafter) and 20 (n = 18 thereafter). Statistical differences in daily water and food intake and body weight were compared weekly using multiple *t*-test with false discovery rate (FDR) correction (**A**–**C**). The difference in survival between the two groups was analyzed by the Mantel–Cox log-rank test (**D**) (χ^2^ = 0.3507, df = 1, *p* = 0.5537). Values are a mean ± SD. * *p* < 0.05.

Hence, throughout the 20-week study, male C57BL/6 mice were given ad libitum either the 3% SP-supplemented water (SP-fed) or the equimolar saline solution (1.6% NaCl-containing water; saline-fed). Daily water intake did not differ significantly between the two groups (Figure 1A). However, statistically trivial differences in daily food consumption were observed from week 4 onwards (Figure 1B). While both groups exhibited a steady increase in daily food intake as they aged, the amount of increase was slightly lower in the SP-fed mice. Similarly, both groups showed gradual weight gain over time, although the rate was slightly slower in the SP-fed mice (Figure 1C). Regarding mortality, over the 20-week duration of the study, which began with 40 mice (20 per group), three deaths occurred: one in the saline-fed mice (week 16) and two in the SP-fed mice (weeks 5 and 20). These results support no statistically significant differences in the survival rates between the groups (Figure 1D).

These findings demonstrate that the long-term ad libitum administration of 3% SP via drinking water may be a safe and well-tolerated chronic high-dose regimen for continuously supplying exogenous pyruvate to mice.

### 3.2. In Vivo Proton Magnetic Resonance Spectroscopy (^1^H-MRS)-Assisted Detection of Metabolic Neurochemicals in the Brain

**Figure 2:**
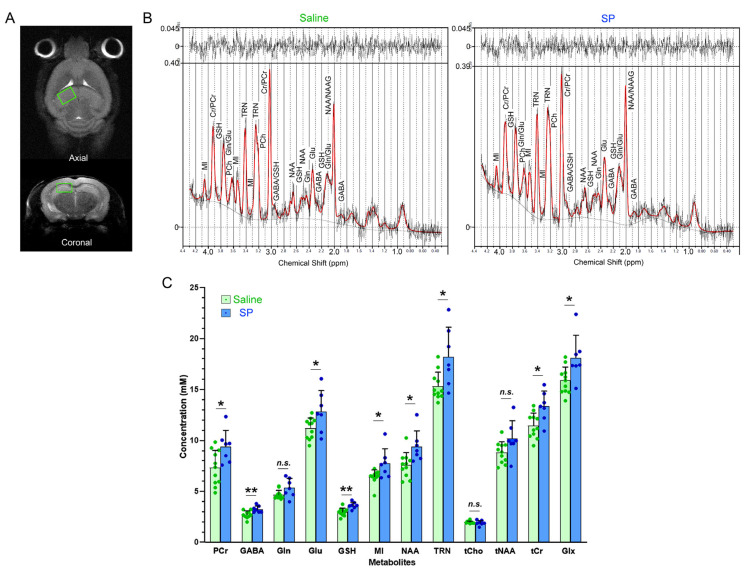
Effects of long-term administration of exogenous pyruvate via sodium pyruvate (SP)-supplemented drinking water on neurochemical metabolism in the mouse brain. After normal male C57BL/6 mice continued to ingest either 3% SP-supplemented water (SP-fed; blue) or 1.6% saline solution (saline-fed; green) ad libitum for 20 weeks, alterations in essential neurochemicals in the hippocampus were analyzed using in vivo proton MRS. Cerebral concentrations (mM) of neurochemical metabolites with Cramér-Rao Lower Bound (CRLB) values below 20% ((**C**), and Supplementary Table S1) were quantified from proton MR spectra profiles obtained in the left hippocampus ((**A**), green square) of the saline-fed (left spectra in (**B**), n = 11) or the SP-fed (right spectra in (**B**), n = 7) normal mice. Values are a mean ± SD. Statistical significance was evaluated using an unpaired *t*-test. * *p* < 0.05 and ** *p* < 0.01, and *n.s.*, not significant (*p* > 0.05). PCr, phosphocreatine; GABA, γ-aminobutyric acid; Gln, glutamine; Glu, glutamate; GSH, glutathione; MI, myo-inositol; NAA, N-acetylaspartate; TRN, taurine; tCho, total choline (glycerophosphocholine + phosphorylcholine); tNAA, total NAA (NAA + N-acetylaspartylglutamate); tCr, total creatine (creatine + phosphocreatine); Glx, glutamate and glutamine complex (Glu + Gln).

The metabolic neurochemical profiles in the mouse hippocampus, the brain region which is primarily affected by epileptic seizure insults, were noninvasively evaluated using in vivo proton MRS at the 20th week of the 3% SP supplementation. Using the typical proton MR spectra from the left hippocampal area (Figure 2A,B), we quantified the absolute concentrations (mM) of the metabolic neurochemicals. Only measurements with a CRLB value of less than 20% [20] were included in the subsequent statistical comparisons (Supplementary Table S1, and Figure 2C).

Compared to the saline-fed control mice, the SP-fed mice exhibited evident increases in PCr, GABA, Glu, GSH, MI, NAA, TRN, tCr, and Glx. Although the Gln, tCho, and tNAA levels were also elevated in the SP-fed mice, these changes were only marginally or not statistically significant.

These results suggest that the long-term ad libitum pyruvate supplementation may sustain significant alterations in the metabolic neurochemical profiles of hippocampal tissues in normal mice.

### 3.3. Liquid Chromatography–Tandem Mass Spectrometry (LC-MS/MS)-Assisted Detection of Energy Metabolites in the Brain

**Figure 3:**
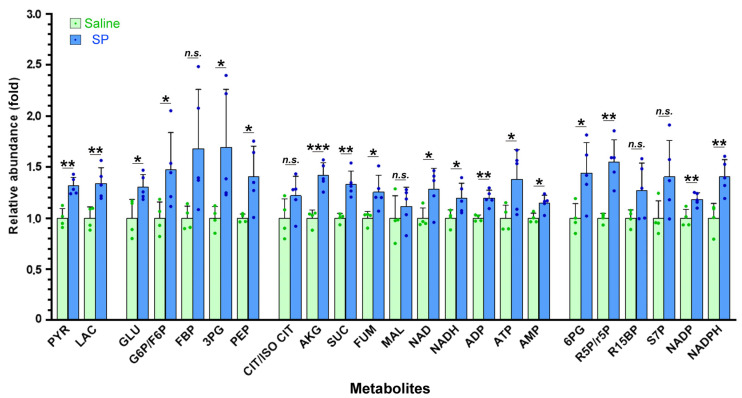
Effects of long-term administration of exogenous pyruvate via sodium pyruvate (SP)-supplemented drinking water on energy metabolism in the mouse brain. After normal male C57BL/6 mice continued to drink either 3% SP-supplemented water (SP-fed) or 1.6% saline solution (saline-fed) for 20 weeks, changes in energy metabolites in the brain tissues, were analyzed using LC-MS/MS-assisted targeted metabolomics. Levels of energy substrate, intermediate, and end product metabolites involved in glycolysis, gluconeogenesis, the pentose phosphate pathway (PPP), the tricarboxylic acid (TCA) cycle, and oxidative phosphorylation (OXPHOS) in the brains of SP-fed mice (blue, n = 5) are depicted relative to those in saline-fed control mice (green, n = 4) as fold-times. Values are a mean ± SD. Statistical significance was evaluated using an unpaired *t*-test. * *p* < 0.05, ** *p* < 0.01, and *** *p* < 0.001, and *n.s.*, not significant (*p* > 0.05). PYR, pyruvate; LAC, lactate; GLU, glucose; G6P/F6P, glucose-6-phosphate/fructose-6-phosphate; FBP, fructose-1,6-bisphosphate; 3PG, 3-phosphoglycerate; PEP, phosphoenolpyruvate; CIT/ISO CIT, citrate/isocitrate; AKG, α-ketoglutarate; SUC, succinate; FUM, fumarate; MAL, malate; NAD, oxidized form of nicotinamide adenine dinucleotide; NADH, reduced form of nicotinamide adenine dinucleotide; ADP, adenosine diphosphate; ATP, adenosine triphosphate; AMP, adenosine monophosphate; 6PG, 6-phosphogluconate; R5P/r5P, ribulose-5-phosphate/ribose-5-phosphate; R15BP, ribose 1,5-bisphosphate; S7P, sedoheptulose-7-phosphate; NADP, oxidized forms of nicotinamide adenine dinucleotide phosphate; NADPH, reduced form of nicotinamide adenine dinucleotide phosphate.

To comprehensively evaluate the impact of the 20-week 3% SP supplementation on cerebral pyruvate, glucose, and energy metabolism, encompassing glycolysis, gluconeogenesis, the mitochondrial TCA cycle, OXPHOS, and the PPP, we conducted LC-MS/MS analysis of the brain tissues comprising the hippocampus and cortex (Figure 3).

Our priority was to determine whether and how the long-term exogenous pyruvate supplementation via drinking water influences cerebral pyruvate levels. The SP-fed mice exhibited significantly higher levels of pyruvate (PYR) in their brains than the saline-fed mice. Correspondingly, the broad enhancement of glucose and energy metabolism was observed in the SP-fed mice, as nearly all intermediate metabolites and the end products across these pathways were significantly elevated. Specifically, the metabolites involved in glycolysis and gluconeogenesis, including glucose (GLU), glucose-6-phosphate/fructose-6-phosphate (G6P/F6P), fructose-1,6-bisphosphate (FBP), 3-phosphoglycerate (3PG), phosphoenolpyruvate (PEP), and lactate (LAC), were significantly or moderately higher.

Regarding mitochondrial metabolism, the α-ketoglutarate (AKG), succinate (SUC), and fumarate (FUM) levels were significantly increased, whereas the citrate/isocitrate (CIT/ISO CIT) and malate (MAL) showed modest increases with lower statistical significance. The adenine nucleotides and their cofactors also increased; the oxidized and reduced forms of nicotinamide adenine dinucleotide (NAD and NADH), adenosine diphosphate (ADP), adenosine triphosphate (ATP), and adenosine monophosphate (AMP) were all elevated in the SP-fed mice. Furthermore, they showed significantly higher levels of the PPP components, including 6-phosphogluconate (6PG), ribulose-5-phosphate/ribose-5-phosphate (R5P/r5P), and both the oxidized and reduced nicotinamide adenine dinucleotide phosphate (NADP and NADPH). The levels of sedoheptulose-7-phosphate (S7P) and ribose 1,5-bisphosphate (R15BP) also increased, although the changes were not statistically significant.

These findings indicate that long-term exogenous pyruvate supplementation may enhance pyruvate utilization in the brain, activating multiple pathways across glucose and energy metabolism.

### 3.4. Antiseizure Effects of Long-Term Pyruvate Supplementation on Kainate-Induced Status Epilepticus

Following 20 weeks of continuous SP supplementation, the mice received an intraperitoneal injection of 40 mg/kg KA to induce severe, protracted SE without anticonvulsant intervention [17,18].

**Figure 4:**
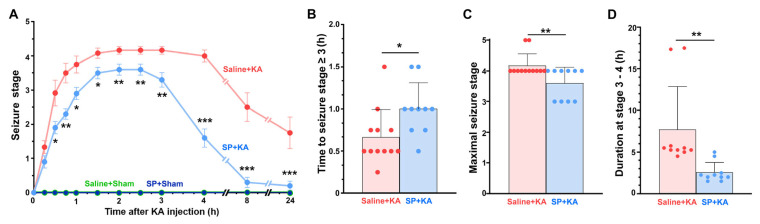
Antiseizure effects of long-term administration of exogenous pyruvate via sodium pyruvate (SP)-supplemented water against kainate (KA)-induced status epilepticus (SE). Mice maintained on either saline (saline-fed) or 3% SP-supplemented drinking water (SP-fed) for 20 weeks were intraperitoneally administered 40 mg/kg KA. (**A**) Seizure severity was behaviorally assessed at the indicated time points. The saline-fed sham (Saline + Sham; green, n = 7) and SP-fed sham (SP + Sham; dark blue, n = 5) control mice exhibited normal behavior and remained at stage 0 throughout the evaluation. In contrast, seizure activity and progression were observed in both the saline-fed/KA-injected (Saline + KA; red, n = 12) and SP-fed/KA-injected (SP + KA; sky blue, n = 10) mice, between which significant differences emerged. Two of the 12 saline-fed/KA-injected mice reached stage 5 and died, whereas none of the 10 SP-fed/KA-injected animals showed such mortality (**C**). (**B**–**D**) Behavioral assessments included the latency to first manifest seizure episodes corresponding to stage ≥ 3 (**B**), the maximal seizure stage reached (**C**), and the duration spent in stages 3 and 4 (**D**). Values are a mean ± SD. Statistical differences between the saline-fed/KA-injected (Saline + KA) and SP-fed/KA-injected (SP + KA) mice were determined using multiple *t*-test with false discovery rate (FDR) correction (**A**) or an unpaired *t*-test (**B**–**D**). * *p* < 0.05, ** *p* < 0.01, and *** *p* < 0.001.

Sham administration using normal saline instead of KA caused no seizure activity or abnormal behaviors in the saline-fed and SP-fed mice, with all the sham mice remaining at stage 0 (Figure 4A). However, within 15 min of the KA injection, most KA-injected mice exhibited seizure behavioral severity corresponding to stage 1. Subsequently, in the saline-fed/KA-injected mice, seizure episodes progressed more rapidly, reached greater severity, and lasted longer with a delayed recovery compared to the SP-fed/KA-injected mice (Figure 4). Two of the 12 saline-fed/KA-injected mice died from KA-induced epileptic injury, corresponding to stage 5, whereas all survivors progressed to stage 4 (Figure 4C). Contrastingly, all 10 SP-fed/KA-injected mice survived KA-induced SE after reaching stage 3 or 4 (Figure 4C) and recovered more rapidly (Figure 4A,D). Therefore, the SP-fed/KA-injected mice exhibited lower susceptibility, higher survival rates, and greater resilience to KA-induced SE than the saline-fed/KA-injected mice.

These findings may support the potential of long-term ad libitum administration of exogenous pyruvate via 3% SP-supplemented drinking water as an antiseizure regimen to prevent or mitigate seizure activity in KA-injected mice.

### 3.5. Neuroprotective Effects of Long-Term Pyruvate Supplementation on Kainate-Induced Status Epilepticus

**Figure 5:**
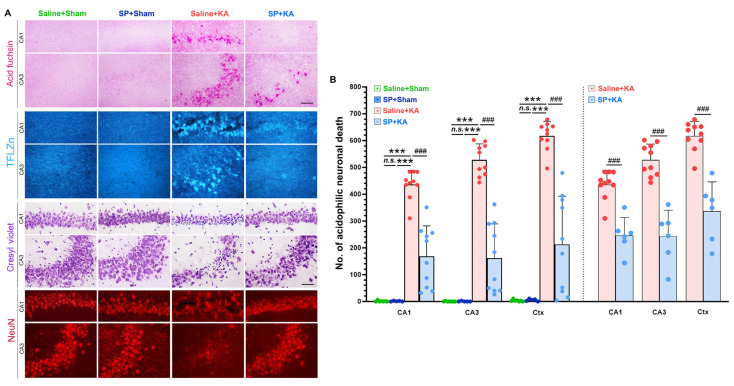
Neuroprotective effects of long-term supplementation of sodium pyruvate (SP) in drinking water against kainate (KA)-induced status epilepticus (SE). Mice maintained on either saline (saline-fed) or 3% SP-supplemented drinking water (SP-fed) for 20 weeks were intraperitoneally administered 40 mg/kg KA. (**A**) In mice that experienced stage 4 of seizure severity, degenerating and surviving neurons in the hippocampal CA1 and CA3 pyramidal layers were visualized using acid fuchsin, TFLZn, or cresyl violet staining, as well as NeuN immunofluorescent labeling. Representative microphotographs are shown for the saline-fed (Saline + Sham) and SP-fed (SP + Sham) control mice, as well as the saline-fed/KA-injected (Saline + KA) and SP-fed/KA-injected (SP + KA) mice. Magnification, 400×; scale bars, 50 μm. (**B**) Dead or degenerating neurons, identified by pink-colored acidophilicity ((**A**), 1st and 2nd rows), were counted across the hippocampal CA1 and CA3 pyramidal and the cortical (Ctx) regions of both hemispheres on five coronal tissue sections collected every 200 μm from bregma −1.3 mm. The comparison was performed among all tested groups (left graph) and specifically in animals that experienced KA-induced SE at stage 4 (right graph), including the saline-fed control (Saline + Sham; green, n = 7) SP-fed (SP + Sham; dark blue, n = 5) sham mice, and the saline-fed/KA-injected (Saline + KA; red, n = 10), and SP-fed/KA-injected (SP + KA; sky blue, n = 10 or 6) mice. Values are a mean ± SD. Statistical differences between groups were determined using one-way ANOVA followed by Tukey’s HSD multiple comparisons test (left panel) or an unpaired *t*-test (right panel). ***^,###^ *p* < 0.001, and *n.s.*, not significant (*p* > 0.05).

To examine neuronal death following severe KA-induced SE, brain sections were stained with acid fuchsin, TFLZn, and cresyl violet to identify acidophilicity, free zinc accumulation, and pyknosis, respectively (Figure 5A). The extent of neuronal death was measured by counting acidophilic dead neurons in the hippocampal CA1 and CA3 pyramidal layers and the cerebral cortex (Figure 5B). While neuronal death was scarce in the brain tissues of the saline-fed and SP-fed sham mice, numerous dead neurons appeared in both the saline-fed/KA-injected and SP-fed/KA-injected mice, between which significant differences were observed, with the latter exhibiting a reduction of over 60% compared to the former. Even when evaluating animals exclusively at seizure stage 4, accounting for the potential influence of the maximal seizure severity experienced by the mice on neuronal death [17,18], the SP-fed/KA-injected mice still showed significant reductions, which were approximately 45–55% lower than in the saline-fed/KA-injected mice (Figure 5B).

Consistent with these assessments, immunofluorescence staining for NeuN, a neuronal marker, revealed a significant depletion of NeuN-immunopositive (+) pyramidal neurons in the hippocampi of the saline-fed/KA-injected mice compared to the sham mice, in which the NeuN(+) pyramidal neurons remained intact and prevalent. However, this loss was significantly attenuated in SP-fed/KA-injected mice, even when experiencing seizure stage 4 (Figure 5A). Conversely, pyknotic bodies, cell death markers visualized via cresyl violet staining (Figure 5A), were markedly elevated in the saline-fed/KA-injected mice, but substantially reduced in the SP-fed/KA-injected mice.

These findings may underscore the neuroprotective potential of long-term ad libitum 3% SP administration via drinking water, demonstrating its efficacy in preserving neuronal survival and preventing or attenuating neurodegeneration, even after the mice suffered from severe KA-induced SE.

Notably, in the saline-fed mice that had experienced stage 4 of KA-induced SE, the majority of dead or dying neurons, as characterized by their acidophilicity and pyknosis, emitted intense TFLZn fluorescence (Figure 5A), indicating a pathological accumulation of chelatable zinc within their somata. However, zinc accumulation was significantly suppressed in the SP-fed/KA-injected mice, albeit experiencing the same stage 4 of KA-induced SE, where the intensity of the TFLZn fluorescence was markedly attenuated, often falling below the neurotoxic threshold or being nearly abolished.

These findings suggest that the long-term ad libitum supplementation may effectively prevent or reduce zinc-induced neuronal death following KA-induced SE, likely by preserving intracellular zinc homeostasis [9,10].

### 3.6. Modulation of Neuroprotein Profiles by Long-Term Pyruvate Supplementation Following Kainate-Induced Status Epilepticus

**Figure 6:**
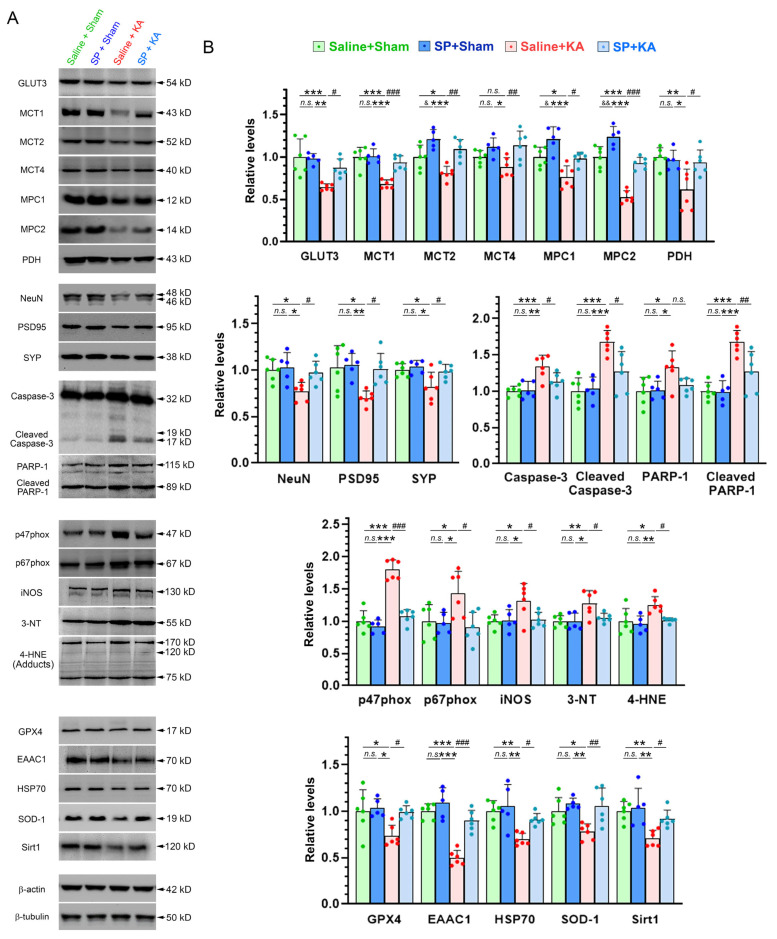
Effects of long-term drinking of sodium pyruvate (SP)-supplemented water on the expression of pyruvate metabolic, neurosynaptic structural and functional, neuropathological, and neuroprotective proteins in the brains of mice with kainate (KA)-induced status epilepticus (SE). (**A**) Representative immunoblots demonstrate the expression levels of proteins related to cerebral glucose and pyruvate transport and metabolism (GLUT3, MCT1, MCT2, MCT3, MPC1, MPC2, and PDH), neuronal and synaptic structure and activity (NeuN, PSD95, and SYP), neuronal death (pro and cleaved caspase-3 and PARP-1), cellular oxidation (p47phox, p67phox, iNOS, 4-HNE, and 3-NT), and antioxidative or neuroprotective activity (GPX4, EAAC1, HSP70, SOD-1, and Sirt1) in the cerebral tissues of the saline-fed control (Saline + Sham) and SP-fed sham (SP + Sham) mice, as well as the saline-fed/KA-injected (Saline + KA), and SP-fed/KA-injected (SP + KA) mice that survived stage 3 or 4 of SE. β-actin or β-tubulin was used as internal loading controls. (**B**) Densitometric quantification of specific protein levels, normalized to β-actin or β-tubulin, in the SP-fed sham mice (SP + Sham; dark blue, n = 5), saline-fed/KA-injected mice (Saline + KA; red, n = 6), and SP-fed/KA-injected mice (SP + KA; sky blue, n = 6) relative to the saline-fed sham controls (=1.0: Saline + Sham; green, n = 6). Bars represent the mean ± SD from triplicate immunoblots. One-way ANOVA followed by Tukey’s HSD multiple comparisons test was used to determine statistical significance. *^,#,&^ *p* < 0.05, **^,##,&&^ *p* < 0.01, and ***^,###^ *p* < 0.001, and *n.s.*, not significant (*p* > 0.05), in which asterisks (*), sharps (#) and ampersands (&) denote comparisons of the saline-fed sham control vs. the saline-fed/KA-injected mice, the saline-fed/KA-injected vs. the SP-fed/KA-injected mice, and the saline-fed sham control vs. the SP-fed sham mice, respectively.

To elucidate the effects of long-term 3% SP supplementation on the neuropathological and neuroprotective profiles of the mouse brain during KA-induced SE, semi-quantitative immunoblotting analyses were conducted on various molecular signaling proteins in cerebral tissues obtained from the mice that had experienced stage 3 or 4 of KA-induced SE (Figure 6 and Supplementary Figure S1).

Given that the primary objective was to assess the impact of exogenous pyruvate administered via 3% SP-supplemented water on the brain, we were initially interested in the proteins involved in cerebral pyruvate transport and metabolism. Compared to the saline-fed sham controls, the SP-fed sham mice exhibited significant increases in the protein levels of MCT2, MPC1 and MPC2. In contrast, no significant differences were detected in the expressions of GLUT3, MCT1, MCT4, and PDH between the two sham groups. Following KA-induced SE, the saline-fed/KA-injected mice showed a marked reduction in the levels of these proteins relative to the sham mice. However, the SP-fed/KA-injected mice exhibited significantly higher levels of these metabolic proteins than the saline-fed/KA-injected mice, with values approaching those of the sham mice.

Except for MCT2, MPC1, and MPC2, no significant differences were observed in the expression levels of most tested proteins between the saline-fed and SP-fed sham mice. Following KA-induced SE, however, proteins associated with neuronal and synaptic integrity or function, including NeuN, PSD95, and SYP, were significantly downregulated in the saline-fed/KA-injected mice, whereas their expression was robustly preserved or upregulated in the SP-fed/KA-injected mice. Conversely, pro-apoptotic markers, including caspase-3, PARP-1, and their cleaved forms c-caspase-3 and c-PARP-1, were elevated in the saline-fed/KA-injected mice but markedly reduced in the SP-fed/KA-injected mice.

Whereas in the saline-fed/KA-injected mice the levels of the NADPH oxidase subunits p47phox and p67phox, the reactive oxygen species (ROS) inducer iNOS, and the oxidative stress markers 3-NT and 4-HNE were increased, those of the antioxidants GPX4, EAAC1, and SOD-1, as well as the neuroprotective proteins HSP70 and Sirt1 were decreased. However, in the SP-fed/KA-injected mice, these alterations were effectively reversed, reducing the levels of p47phox, p67phox, iNOS, 3-NT, and 4-HNE, and significantly elevating the levels of GPX4, EAAC1, SOD-1, HSP70, and Sirt1, even though they have experienced the corresponding severe stages of KA-induced SE.

These immunoblotting results, along with the aforementioned histological findings (Figure 5), demonstrate that long-term ad libitum 3% SP supplementation may sustain or facilitate multifaceted neuroprotective properties against KA-induced SE in the mice.

### 3.7. Effects of Exogenous Pyruvate on Neurobehavioral Performances in Mice Subjected to Kainate-Induced Status Epilepticus

**Figure 7:**
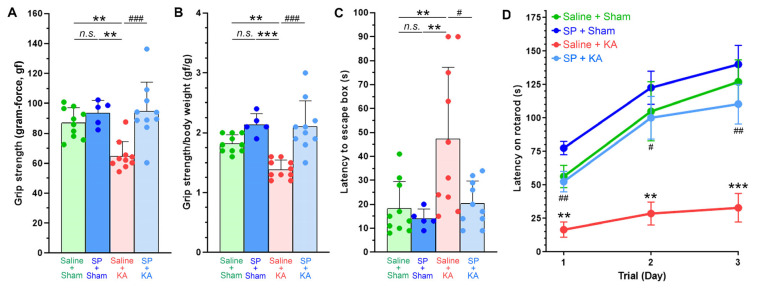
Effects of long-term administration of exogenous pyruvate via 3% sodium pyruvate (SP)-supplemented drinking water on motor and neuromuscular behaviors in mice with kainate (KA)-induced status epilepticus (SE). Saline-fed control (Saline + Sham; green, n = 10), SP-fed (SP + Sham; dark blue, n = 5) sham, saline-fed/KA-injected (Saline + KA; red, n = 10), and SP-fed/KA-injected (SP + KA; sky blue, n = 10) mice were subjected to grip strength (**A**,**B**), balance beam (**C**), and rotarod (**D**) tests to assess their performance in balancing, coordination, and control of neuromuscular or motor activities. (**A**,**B**) Grip strength (gram-force, gf) is shown without (**A**) and with (**B**) normalization to grams of body weight. Values are the means of triplicate trials conducted consecutively. (**C**) The mean time (second, s) taken to traverse the beam and enter the opposite escape chamber in three repeated trials represented the escape latency of the tested animal. (**D**) The mean latency time (second, s) to walk on the rotating rod without falling in the rotarod test, performed daily in triplicate, was traced for three consecutive days. Statistical comparisons among the groups were performed using one-way ANOVA followed by Tukey’s HSD multiple comparisons test, with values denoting a mean ± SD (**A**–**C**) or a mean ± SEM (**D**). Asterisks (*) and sharps (#) indicate significant differences between the saline-fed sham control mice and the saline-fed/KA-injected mice, and between the saline-fed/KA-injected mice and the SP-fed/KA-injected mice, respectively. ^#^ *p* < 0.05, **^,##^ *p* < 0.01, and ***^,###^ *p* < 0.001, and *n.s.*, not significant (*p* > 0.05).

Neuromuscular and motor functions were assessed via a four-limb grip strength test (Figure 7A,B). Although not statistically significant, the SP-fed sham mice showed a trend toward slightly increased grip strength compared to the saline-fed sham controls. While the saline-fed/KA-injected mice exhibited significantly reduced grip strength relative to the sham mice, the SP-fed/KA-injected mice demonstrated a significant reversal of grip strength deficits. Thereafter, the balance beam test was employed to evaluate motor coordination, balance, and control (Figure 7C). Performance on the balance beam was comparable between the two sham mice. The SP-fed/KA-injected mice showed enhanced stability and reduced traversing latency compared to the saline-fed/KA-injected mice, restoring performance to levels comparable to those of the sham mice. In the rotarod test (Figure 7D), the saline-fed/KA-injected mice displayed marked motor deficits, as evidenced by the reduced latency to fall and lack of improvement across three daily trials. However, despite suffering from the comparable stage of KA-induced SE, the SP-fed/KA-injected mice exerted a longer latency in the first trial and sustained the performance improvement throughout the testing period, compared to the saline-fed/KA-injected mice. Notably, the SP-fed sham mice exhibited the highest rotarod performance among all tested mice, including the saline-fed sham controls, though this did not always reach statistical significance.

These findings suggest that the long-term ad libitum administration of exogenous pyruvate via 3% SP-supplemented water may prevent or improve neuromuscular and motor impairments in the mice following KA-induced SE.

**Figure 8:**
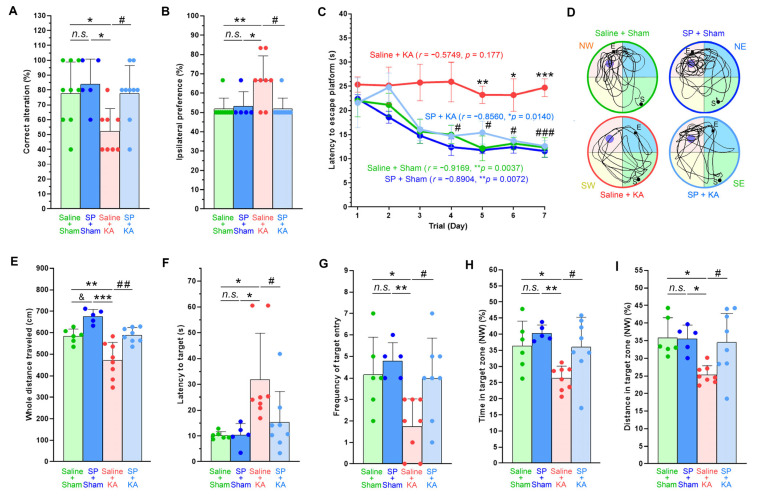
Effects of long-term supplementation of sodium pyruvate (SP) in drinking water on cognitive performance in mice with kainate (KA)-induced status epilepticus (SE). (**A**,**B**) Using a T-maze, the spontaneous alternative side choice test was conducted to evaluate the spatial working memory (**A**) and ipsilateral preferences (**B**) of mice. When the animals could freely choose a side arm to explore for six trials in a row, a choice that differed from the directly preceding trial was considered a correct alternation (%) (**A**), whereas a proclivity to favor any side arm, left or right, was defined as an ipsilateral preference (%) (**B**). Statistical differences among the saline-fed sham control (Saline + Sham; green, n = 9), SP-fed sham (SP + Sham; dark blue, n = 5), saline-fed/KA-injected (Saline + KA; red, n = 8), and SP-fed/KA-injected (SP + KA; sky blue, n = 9) mice were determined using one-way ANOVA followed by Tukey’s HSD multiple comparisons test. (**C**) For the test of spatial learning and memory performance in the Morris water maze (MWM), the saline-fed sham control (Saline + Sham; green, n = 6), SP-fed sham (SP + Sham; dark blue, n = 5), saline-fed/KA-injected (Saline + KA; red, n = 8), and SP-fed/KA-injected (SP + Sham; sky blue, n = 8) mice were trained for three daily trials for 7 days, each beginning in the northeast (NE), southeast (SE), or southwest (SW) quadrant in the pool and lasting up to 60 s, to search for an escape platform hidden beneath the water surface in the northwest (NW) quadrant. The mean value of time (second, s) taken to find and step onto the platform in the daily triplicate trials was quantified as the escape latency of the tested animal. The learning effect of repeated training on escape latency was evaluated using the Pearson linear correlation coefficient (*r*) and correlation significance (*p*). (**D**–**I**) Three hours after the final trial, the mice underwent a free-swim probe test. The mice were required to search for the removed escape platform for 60 s, beginning in the SE quadrant (**D**), to assess their ability to retain spatial reference memory that recalled the previous location of the removed platform (small circular purple area in the NW quadrant). The entire path (**D**) and the whole swimming distance (cm) traveled from the starting point, S, to the ending point, E, in the pool (**E**), the time (second, s) taken for the first touch to the removed platform location (**F**), the frequency with which the mice crossed the vestige of the removed platform (**G**), and the percentages of time spent (**H**) and the distance traveled in the target NW quadrant (**I**) were measured. Values are a mean ± SD. Statistical comparisons between groups were conducted using one-way ANOVA followed by Tukey’s HSD multiple comparisons test. Asterisks (*), sharps (#) and ampersands (&) denote comparisons of the saline-fed sham control vs. the saline-fed/KA-injected mice, the saline-fed/KA-injected vs. the SP-fed/KA-injected mice, and the saline-fed sham control vs. the SP-fed sham mice, respectively. *^,#,&^ *p* < 0.05, **^,##^ *p* < 0.01, and ***^,###^ *p* < 0.001, and *n.s.*, not significant (*p* > 0.05).

To assess spatial working memory of the mice, the spontaneous alternation T-maze test was conducted (Figure 8A). The saline-fed/KA-injected mice exhibited a significantly lower rate of correct alternations than the sham mice, reflecting a high frequency of revisiting the arm chosen in the previous trial. In contrast, the SP-fed/KA-injected mice effectively normalized the correct alternation rates, reaching levels similar to those of the sham mice. Furthermore, whereas the saline-fed/KA-injected mice displayed a marked ipsilateral preference as they chose the identical side arm (right or left) across trial repeats, the SP-fed/KA-injected mice, along with the sham mice, showed no unilateral preference, remaining close to the 50% chance level (Figure 8B).

Spatial learning and memory were assessed using the MWM test for the next seven days, comprising three trials daily (Figure 8C). Over this training period, both the sham mice and the SP-fed/KA-injected mice progressively improved their performance, whereas the saline-fed/KA-injected mice failed to do so. Specifically, from the fourth day of training onward, the former three groups of mice found the hidden platform significantly faster than the saline-fed/KA-injected mice. Three hours after the final training trial, a free swim probe test was performed to evaluate the retention of spatial reference memory in the animals, that is, recalling the previous location of the removed target platform (Figure 8D–I). When estimating the latency to reach the target location for the first time (Figure 8F), the frequency of crossings (Figure 8G), the time spent (Figure 8H), and the distance traveled (Figure 8I) within the target quadrant, the SP-fed/KA-injected mice exhibited improved spatial memory performance comparable to that of the sham mice, despite having experienced KA-induced severe SE. In contrast, the saline-fed/KA-injected mice performed poorly in the probe test. The total swimming distance traveled to locate the removed platform during the probe test was greatest in the SP-fed sham mice, indicating that they swam the most actively and rapidly, followed by the saline-fed sham mice and the SP-fed/KA-injected mice. However, this distance was significantly shorter in the saline-fed/KA-injected mice (Figure 8E).

These results demonstrate that whereas KA-induced SE is comorbid with neurocognitive deficits in animals, the long-term ad libitum administration of exogenous pyruvate may alleviate or improve the cognitive impairments.

## 4. Discussion

Exogenous pyruvate, administered as SP, has demonstrated potent neuroprotection in animal models of cerebral ischemia [9,10], cortical contusion injury [11,12], and hypoglycemia [13]. SP has also been reported to effectively mitigate neuronal loss following epileptic seizures induced by KA or pilocarpine in rats [14,15,16]. These findings support the promising therapeutic potential of exogenous pyruvate in ameliorating neuronal damage in animal models of brain disorders. However, although single high-dose bolus administrations of SP (250–1000 mg/kg, i.p.) developed significant neuroprotection in these animal models, even slight deviations from this established range resulted in a complete loss of efficacy [9,10]. The acute robust infusion of large amounts of exogenous pyruvate has been reported to cause SE, respiratory arrest, and even death, rather than providing neuroprotection [22]. While exogenous pyruvate elicits potent neuroprotection at relatively higher dose levels, its efficacy window is narrow and varies across different disease models with respect to the administered dose, route, timing, and duration [10,23].

This study reveals that, instead of the robust systemic administration of a single large dose of SP, the long-term continuous ad libitum drinking of SP-supplemented water led to sustainable changes in the cerebral content of neurochemical and energy metabolites, and thereby facilitated antiseizure and neuroprotective properties to prevent or mitigate the development and behavioral severity of epileptic seizures and to resist or alleviate neuronal damage, respectively, in mice affected by KA-induced SE.

In vivo proton MRS analysis at the 20th week of ad libitum drinking of 3% SP-supplemented water showed significantly elevated hippocampal concentrations of various metabolic neurochemicals in the SP-fed mice compared to the saline-fed mice, including PCr, tCr [24], GABA [25,26,27,28], Glu [29], Glx [30], GSH [30], MI [31,32], NAA [33,34,35], tNAA [36], and TRN [37,38], all of which are essential for normal or enhanced neuronal physiology, function, bioenergetics, and neuroprotective properties. The magnitude of these increases for most neurochemicals was consistently moderate (110–130% vs. controls), which likely serves to prevent any potential neuropathological damage that could arise from excessive levels. Moreover, these measurements indicate that neurological issues or neuronal injuries arising from the disproportionate overproduction of a specific neurochemical could be effectively counteracted by a coincident increase in its antagonistic counterparts. Specifically, since either an excess of excitatory glutamate (Glu) or a deficiency of inhibitory GABA causes extreme neuronal excitation followed by the occurrence of brain disorders such as epileptic seizures [39], the maintenance of a sustainable excitatory-inhibitory (E/I) balance through the simultaneous increases in both GABA and Glu is essential for normal neuronal activity and for protecting the brain from excitotoxic injury [25,26]. Additionally, numerous prior studies, including proton MRS analysis, have demonstrated the antiseizure and neuroprotective roles of those increased neurochemicals, specifically Cr, PCr [40,41], MI [42,43], NAA [44], and TRN [45], as their cerebral abundance has been reported to correlate negatively with the incidence and symptomatic severity of seizures, as well as with the extent of neuronal loss and associated neurobehavioral deficits in both human patients and animal models of SE.

These findings suggest that the long-term ad libitum pyruvate supplementation may lead to sustainable, moderate increases in the essential metabolic neurochemicals in the brain, continuously underlying neuronal activity and function while offering prophylactic neuroprotection against the subsequent epileptic injury.

To evaluate the effects of the long-term drinking of 3% SP-supplemented water on pyruvate, glucose, and energy metabolism in the brain, mouse cerebral tissues were analyzed using LC-MS/MS-assisted metabolomics. The levels of pyruvate (PYR) and lactate (LAC) in the brains of the SP-fed mice were significantly higher than those in the saline-fed control mice. Regardless of whether exogenous pyruvate, when administered through drinking water, food, gavage, or systemically, reaches the brain directly as pyruvate *per se* or as lactate following the first-pass gastrointestinal or hepatic conversion [46], these findings indicate that the long-term supplementation of exogenous pyruvate can significantly elevate their contents and utilization in the brain [22,47]. Studies have shown that orally administered pyruvate, similar to other monocarboxylates like lactate and ketone bodies, enters the brain *per se* via the monocarboxylate transporters (MCTs) located in the gastrointestinal epithelium and neurovascular unit, where they are directly involved in the cerebral metabolic processes [48,49,50].

Most metabolic intermediates and the end product, ATP, which comprise the TCA cycle and OXPHOS, were significantly or slightly higher in the SP-fed mice, responsive to the enhancement of pyruvate-mediated mitochondrial energy metabolism. The increase in most metabolites involved in glycolysis, gluconeogenesis, and the PPP was also found, representing a broad improvement in cerebral energy, glucose, and pyruvate metabolism in the SP-fed mice. Pyruvate enters the PPP via gluconeogenic conversion to G6P, an intermediate metabolite that facilitates the generation of NADPH and R5P/r5P. Consequently, NADPH provides reducing equivalents for the redox balance and antioxidant GSH regeneration [51,52], thereby serving to maintain the antioxidant capacity in the brain. The current metabolomic findings suggest that the long-term ad libitum intake of 3% SP-supplemented water may continuously supply the brain with pyruvate as an alternative energy source or metabolic mediator, elevating the basal energy level, maintaining the metabolic balance, and enhancing the antioxidant potential in the brain.

Epileptic seizures are a manifestation of intense and sustained hyperexcitability of neurons in the affected regions of the brain, a condition that inevitably demands high energy, primarily supported by increased glucose metabolism. Impairments in glycolytic, gluconeogenic [53], PPP [54], or mitochondrial metabolism [55,56] stimulate or aggravate epileptogenesis and epileptic seizures. Hence, as pyruvate not only serves as the principal mitochondrial energy substrate but also drives gluconeogenesis and the PPP, the long-term continuous supplementation of exogenous pyruvate may compensate for the impaired energy and glucose metabolism in the brain affected by epileptic seizures [57,58].

Indeed, SP as an exogenous pyruvate source has shown significant promise for the prevention and management of epileptic seizures and SE [59,60], which respond to its demonstrated potency in enhancing the neurochemical and energy metabolism in the brain. SP has been reported to moderate seizure phenotypes in rodent models [57,61,62,63]. Clinical trials have previously reported that daily oral SP administration over several months consistently suppressed epileptic seizures in pediatric patients with Leigh syndrome, a progressive neurodegenerative mitochondrial disorder characterized by epileptic encephalopathy and intractable generalized convulsive seizures, stemming from the mitochondrial metabolic dysfunction caused by PDH deficiency [64,65]. Subsequently, the chronic oral SP treatment through food, water, or gavage was found to reduce the epileptic phenotypes in rat models of focal and generalized convulsive seizures, as well as in APPswe/PS1dE9 transgenic mice, an animal model of Alzheimer’s disease-associated TLE [57,61]. A single intraperitoneal SP pretreatment also attenuated the severity of KA-induced seizures in mice, and a few days of consuming SP-supplemented water decreased the incidence of spontaneous seizures in Kcna1-null mice, a genetic model of TLE that lacks voltage-gated Kv1.1 channels [62,63].

In the current study, the antiseizure effects of the long-term ad libitum intake of 3% SP-supplemented water on KA-induced SE in mice were assessed by evaluating behavioral symptoms based on the seizure severity classification. First, we observed the absence of seizure activity in both the saline-fed and SP-fed sham mice that received normal saline instead of KA, implying that the long-term ad libitum drinking of 3% SP-supplemented water does not contribute to the induction of spontaneous seizures. When receiving an identical dose of KA, the SP-fed/KA-injected mice demonstrated significantly slower seizure progression, less severe seizure behaviors, shorter seizure durations, faster recovery from seizure episodes, and a higher likelihood of survival than the saline-fed/KA-injected mice. These findings may provide the antiseizure potential of the long-term drinking of SP-supplemented water to confer prevention, resistance, or resilience to epileptic seizures in the brain of KA-induced SE.

The long-term ad libitum drinking of either 3% SP-supplemented water or the equivalent saline solution resulted in negligible neuronal death in the brains, as evidenced by multiple histological markers, including acid fuchsin, TFLZn, and cresyl violet staining, and NeuN immunohistochemical labeling. However, whereas KA-induced SE caused extensive neuronal loss in the saline-fed/KA-injected mice, it was markedly attenuated in the SP-fed/KA-injected animals. Even when evaluating only the animals that reached the highest level of seizure severity (stage 4), as the magnitude of seizure activity can influence the extent of neuronal death [18,66], the SP-fed/KA-injected mice still exhibited significantly less neuronal loss relative to their saline-fed/KA-injected counterparts. Further supporting these neuroprotective effects, immunoblotting results of the cerebral tissues showed that KA-induced SE significantly reduced the neurosynaptic marker proteins, NeuN, PSD95, and SYP levels in the saline-fed/KA-injected mice compared to those in the sham mice, between which the protein levels remained comparable. In contrast, the SP-fed/KA-injected mice maintained protein levels comparable to those of the sham mice and even exhibited slightly higher levels. Conversely, the expression and cleaved activation of the cell death-mediating proteins caspase-3 and PARP-1 were elevated in the saline-fed/KA-injected mice but were markedly reduced in the SP-fed/KA-injected mice. Hence, these findings support the neuroprotective potential [59,60] of the long-term ad libitum drinking of 3% SP-supplemented water to prevent neuronal loss and ensure their survival in the brains of mice with KA-induced SE.

In the brains of the saline-fed/KA-injected mice, numerous neurons exhibited intense TFLZn fluorescence within their perikarya, which coincided with acidophilic or pyknotic neuronal death, indicating that an overload of reactive free zinc, whether resulted from either intracellular release or liberation [67,68] or synaptic translocation [69], was involved in the neuronal death. In the SP-fed/KA-injected mice, however, the TFLZn fluorescence was attenuated, accompanied by a substantial reduction in the number of TFLZn-fluorescent neurons, implying the prevention or reduction of zinc-induced neuronal death. The results indicate that the long-term administration of SP-supplemented water may confer sustained neuroprotection, enabling brain tissues and neurons to resolve and survive the neurotoxic sequelae of zinc accumulation following KA-induced SE, consistent with the effects previously elicited by a single large systemic SP administration in a variety of rodent models of brain injury, including epileptic seizures, where neuronal death responded to excessive intraneuronal zinc loads [67,68,69]. Previous studies have proposed that an intracellular zinc overload instigates the depletion of NAD and ATP via the activation of PARP-1, ultimately resulting in neuronal death [70,71], whereas these detrimental changes have been eliminated by exogenous pyruvate in various models of neuronal injury [70,72]. Consistent with these established mechanisms, this study found a substantial reduction in both intraneuronal zinc accumulation and the PARP-1 activation in the brains of the SP-fed/KA-injected mice, concomitant with the probable restoration of the NAD and ATP levels.

While the principal neuronal energy substrate glucose is transported into neurons via GLUT3 [73], exogenously supplied or endogenously generated pyruvate gains access to the brain and neurons through neurovascular MCT interconnections comprising endothelial and glial MCT1, glial MCT4, and neuronal MCT2 [74,75]. Subsequently, pyruvate enters the mitochondria through the MPC, where it is converted into acetyl-CoA by PDH to fuel the TCA cycle and OXPHOS for ATP synthesis [76]. While these metabolic transporters and enzymes are crucial for energy production and the synthesis of metabolic neurochemicals via pyruvate metabolism, aberrant alterations in their expression or activity are implicated in neuronal damage during epileptic seizures [75]. This study revealed that the levels of GLUT3, MCTs, MPC, and PDH in the brains of the saline-fed/KA-injected mice were reduced compared to those in the saline-fed and SP-fed sham mice. In contrast, the SP-fed/KA-injected mice maintained significantly higher levels of these proteins, even after undergoing KA-induced SE. Notably, the SP-fed sham mice exhibited higher levels of MCT2, MPC1, and MPC2 proteins, accompanied by a greater abundance of essential neurochemical and energy metabolites, compared to the saline-fed sham controls. Collectively, these findings demonstrate that the long-term continuous drinking of 3% SP-supplemented water may lead to sustained elevation of the pyruvate transporters and metabolic enzymes in the brain. This represents an adaptive preconditioning response to a long-term continuous and increased supply of exogenous pyruvate, facilitating sufficient pyruvate and glucose utilization to augment neurochemical and energy metabolism in the brain.

In vivo proton MRS and energy metabolomics revealed that the SP-fed mice exhibited an augmented antioxidant defense capacity in the brain, as evidenced by elevated levels of GSH, TRN, NADPH, LAC, and PYR, the metabolites which are crucial for preventing oxidative damage and maintaining the redox balance. Previous studies have consistently demonstrated that the neuroprotective properties of SP arise from the role of pyruvate as an effective ROS scavenger and potent antioxidant [7,8]. Therefore, this study investigated whether the long-term SP supplementation via drinking water facilitates antioxidative neuroprotection during KA-induced SE. Following KA-induced SE, the brains of the saline-fed/KA-injected mice showed significantly elevated levels of oxidative stress markers, iNOS, 4-HNE, and 3-NT, as well as the p47phox and p67phox subunits of the ROS-producing enzyme NADPH oxidase and the neuronal death-mediating proteins cleaved PARP-1 and caspase-3. In contrast, these protein levels in the SP-fed/KA-injected mice were comparable to or even lower than those found in the sham animals. These findings are consistent with those of earlier studies demonstrating that oxidative stress is implicated not only in epileptogenesis and epileptic seizures but also in neuronal loss following seizure episodes [77,78]. In these cases, neuronal oxidation provokes the overactivation of PARP-1, leading to energy failure through the depletion of ATP and NAD+ and inhibition of glycolysis [79,80], or activates apoptotic caspase-3 [81]. This ultimately augments neuronal death, whereas exogenous pyruvate can mitigate oxidative stress to curtail such neurodegenerative signals [16,63,80]. Furthermore, the levels of the antioxidant enzymes GPX4 [82], EAAC1 [83,84], and SOD-1 were reduced in the saline-fed/KA-injected mice but elevated in the SP-fed/KA-injected mice, with similar changes observed in the neuroprotective proteins HSP70 [14,85,86] and Sirt1 [87,88,89], supporting the molecular mechanism underlying the antioxidative neuroprotection afforded by the continuous pyruvate supplementation. Collectively, these findings support the contention that exogenous pyruvate provided via long-term administration of SP-supplemented water may effectively reinforce the antioxidant defense capacity in the brain, conferring significant neuroprotection against oxidative damage during KA-induced SE.

The findings of this study corroborate previous reports that the potent protective effects of exogenous pyruvate, intraperitoneally administered as SP, on neuronal injuries in various animal disease models stem from its multifaceted neuroprotective properties, which include improving energy and neurochemical metabolism, inducing antioxidative and anti-inflammatory responses, suppressing excitotoxicity and zinc toxicity, preventing neuronal death signals, and ultimately preserving neuronal integrity and survival [23,57,63]. Likewise, the long-term ad libitum drinking of SP-supplemented water may serve as an antiseizure and neuroprotective regimen, conferring resistance or resilience to epileptic seizures and preventing neuronal injuries following KA-induced SE.

Epileptic seizures are frequently accompanied by neurobehavioral comorbidities [2,5]. The symptoms and severity of the neurobehavioral deficits vary with the seizure intensity and the affected brain regions [90,91]. Epileptic seizures and their associated comorbidities, which may share underlying structural or functional neuropathies and etiologies in the brain, often emerge concurrently or have reciprocal causal relationships [6,92]. Animal models of chemically induced SE have been instrumental in experimentally exploring the neurobehavioral deficits comorbid to epilepsy [5,93], involving focal cerebral or systemic injections of KA [4,94] or pilocarpine [95,96] into rodents. In the current study, following KA-induced SE, significant impairments in both motor and cognitive performance were observed in the saline-fed/KA-injected mice experienced with spontaneous recurrent seizures. These mice demonstrated motor deficits in the rotarod, balance beam, and grip strength tests, displaying impaired neuromuscular movements such as balance, coordination, and locomotion. They also performed poorly on both the T-maze spontaneous alternation test of spatial working memory and the MWM test of spatial learning and memory, indicating serious cognitive decline. Furthermore, they showed an increased ipsilateral preference in the T-maze side arm selection, which may stem from KA-induced unilateral hemispheric lesions. In contrast, the SP-fed/KA-injected mice exhibited evident improvements in their neurobehavioral performance, performing all motor and cognitive behavioral tests as fluently as the saline-fed sham control mice, even after experiencing seizure episodes at stage 3 or 4. Notably, the SP-fed sham mice showed modest improvement in motor, neuromuscular, and cognitive behavioral performance compared to the saline-fed sham mice, regardless of statistical significance. Consistent with previous studies reporting that exogenous pyruvate administered as an SP ameliorates motor and cognitive impairments in animal models of Alzheimer’s disease [97,98], Parkinson’s disease [23], severe hypoglycemia [13], traumatic brain injury [99,100], and aging [97], these findings suggest that the long-term continuous ad libitum administration of SP-supplemented water may prevent and improve the neurobehavioral comorbidities associated with epileptic seizures.

Ultimately, the antiseizure and neuroprotective potentials elicited through metabolic enhancement in the animal model of KA-induced SE following long-term exogenous pyruvate supplementation are reminiscent of the prophylactic and therapeutic efficacy of ketogenic diets (KDs) against refractory SE or epileptic seizures. Similar to exogenous pyruvate, ketone bodies as monocarboxylates derived from KD gain direct entry to the brain and neurons through MCTs [101], where they participate as alternative energy sources in energy and neurochemical metabolism, leading to multifaceted neuroprotective effects that curtail neuronal oxidation, inflammation and excitotoxicity; ameliorate mitochondrial dysfunction; and prevent neuronal loss [102,103,104]. In parallel, small organic monocarboxylic short-chain fatty acids (e.g., acetate, propionate, butyrate, and valproate) reportedly exert antiseizure and neuroprotective properties through mechanisms similar to those mentioned above [105,106].

## 5. Conclusions

In conclusion, the long-term continuous administration of exogenous pyruvate via 3% SP-supplemented water may establish metabolic preconditioning in the brain, which sustains enhanced levels of essential neurochemical and energy metabolites, thereby providing comprehensive neuroprotection against seizure activity, neuropathological injury, and associated neurobehavioral deficits in mice following KA-induced SE. Additionally, to corroborate these findings and validate their translational significance, further studies involving larger animal cohorts of both sexes and more diverse animal models are warranted, with careful attention to minimizing potential confounders in the longitudinal and comprehensive animal experimentation.

## Data Availability

Data will be available upon reasonable request to the corresponding author.

## Supplementary Materials

The following supporting information can be downloaded at: https://www.mdpi.com/article/10.3390/biom16060805/s1, Figure S1: Collection of original blots used for Figure 6A; Table S1: Concentrations and CRLB values of metabolic neurochemicals measured using in vivo ^1^H-MRS in the hippocampus of the normal mice fed either saline solution or 3% sodium pyruvate (SP)-supplemented water.

## Abbreviations

The following abbreviations are used in this manuscript:

^1^H-MRS	proton magnetic resonance spectroscopy
3-NT	3-nitrotyrosine
4-HNE	4-hydroxynonenal
ADP	adenosine diphosphate
AMP	adenosine monophosphate
ATP	adenosine triphosphate
Cr	creatine
CRLBs	Cramér-Rao lower bounds
EAAC1	excitatory amino acid carrier 1
GABA	γ-aminobutyric acid
Gln	glutamine
GLU	glucose
Glu	glutamate
GLUT3	glucose transporter 3
Glx	glutamate and glutamine complex
GPX	glutathione peroxidase
GSH	glutathione
HSP70	heat shock protein 70
iNOS	inducible nitric oxide synthase
KA	kainate
KD	ketogenic diets
LC-MS/MS	liquid chromatography–tandem mass spectrometry
LCModel	Linear Combination Model
MCT	monocarboxylate transporter
MI	myo-inositol
MPC	mitochondrial pyruvate carrier
MRI	magnetic resonance imaging
MWM	Morris water maze
NAA	N-acetylaspartate
NAAG	N-acetylaspartylglutamate
NAD	oxidized form of nicotinamide adenine dinucleotide
NADH	reduced form of nicotinamide adenine dinucleotide
NADP	oxidized forms of nicotinamide adenine dinucleotide phosphate
NADPH	reduced form of nicotinamide adenine dinucleotide phosphate
NeuN	neuronal nuclei
OXPHOS	oxidative phosphorylation
PARP-1	poly(ADP-ribose) polymerase-1
PCr	phosphocreatine
PDH	pyruvate dehydrogenase
PPP	pentose phosphate pathway
PSD95	postsynaptic density protein 95
SE	status epilepticus
Sirt1	sirtuin 1
SOD-1	superoxide dismutase-1
SP	sodium pyruvate
SYP	synaptophysin
TRN	taurine
TCA	tricarboxylic acid
TFLZn	N-(6-methoxy-8-quinolyl)-p-carboxybenzoylsulphonamide
TLE	temporal lobe epilepsy
tCr	total creatine
tNAA	total N-acetylaspartate
