# Supplementary material for: Continuous Pyruvate Supplementation Enhances Neuroprotective Resilience Against Kainate-Induced Status Epilepticus Through Metabolic Preconditioning
Source: Biomolecules. 2026 May 29;16(6):805. doi: 10.3390/biom16060805 (PMC13297230; doi:10.3390/biom16060805)
Supplement: Supplementary file 1 [file biomolecules-16-00805-s001.zip › Figure S1(Revised).pdf]

biomolecules

MDPI

Article

Continuous Pyruvate Supplementation Enhances Neuroprotective Resilience Against Kainate-Induced Status Epilepticus through Metabolic Preconditioning

Yong Jae Cho [1,2], Soo Jin Lee [1,2], Yuna Kim [1,2], Yeeun Kim [1,2], Seog-Young Kim [1,3], Kyunggon Kim [1,3,4], Dong-Cheol Woo [1,3], Hyun Ju Yoo [1,3,4] and Joo-Yong Lee [1,3,5,*]

1. Asan Institute for Life Sciences, Asan Medical Center, Seoul 05505, Republic of Korea
2. Department of Medical Science, University of Ulsan College of Medicine, Seoul 05505, Republic of Korea
3. Department of Convergence Medicine, Asan Medical Center, Seoul 05505, Republic of Korea
4. Department of Digital Medicine, University of Ulsan College of Medicine, Seoul 05505, Republic of Korea
5. Department of Biochemistry and Molecular Biology, University of Ulsan College of Medicine, Seoul 05505, Republic of Korea

* Correspondence: jlee@amc.seoul.kr; Tel.: +82-2-3010-4143

Supplementary figure S1. Collection of original blots used for Fig. 6

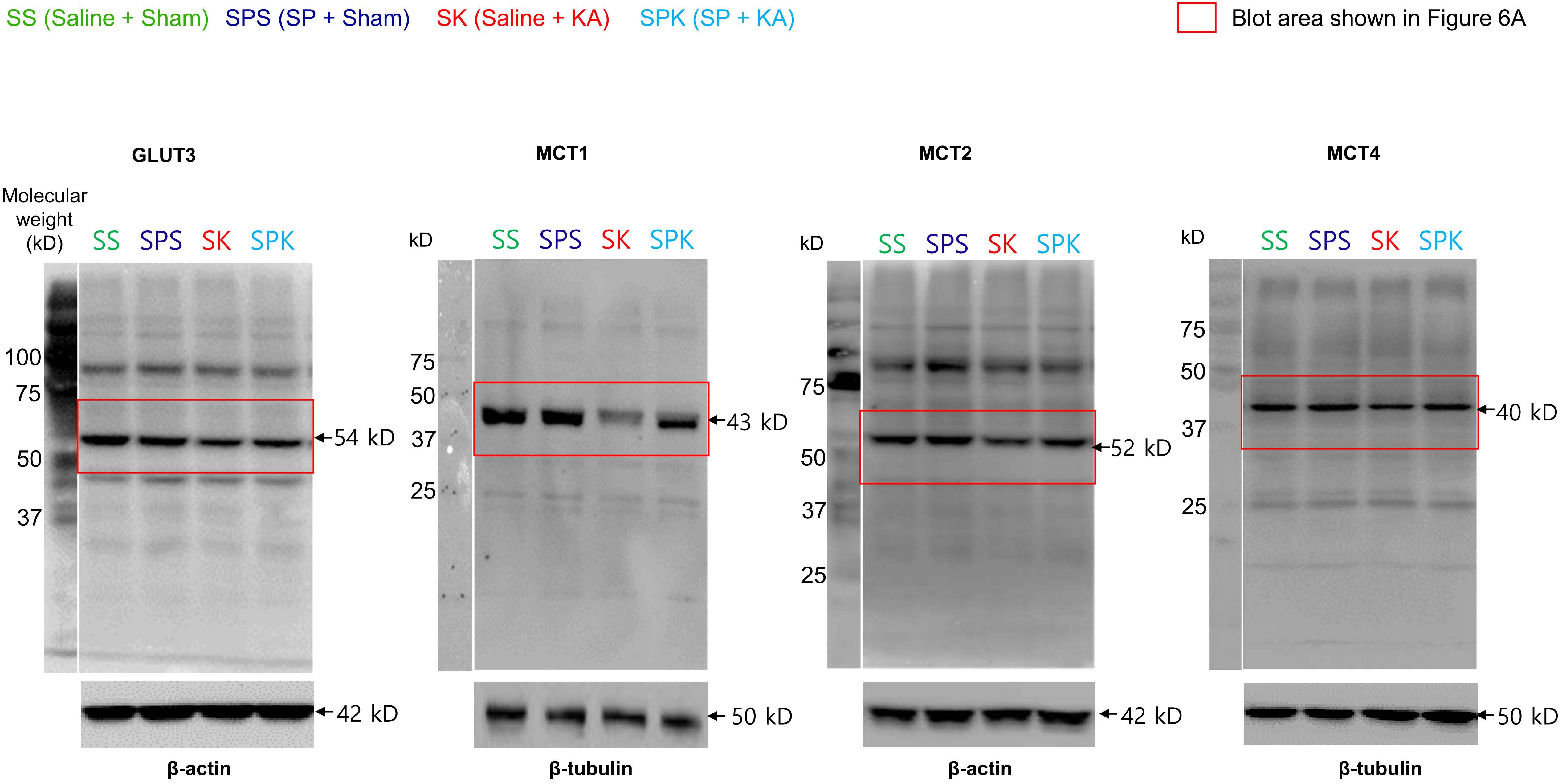

SS (Saline + Sham)
SPS (SP + Sham)
SK (Saline + KA)
SPK (SP + KA)
Blot area shown in Figure 6A
GLUT3
MCT1
MCT2
MCT4
Molecular weight (kD)
SS
SPS
SK
SPK
kD
100
75
50
37
25
54 kD
43 kD
52 kD
40 kD
42 kD
50 kD
β-actin
β-tubulin

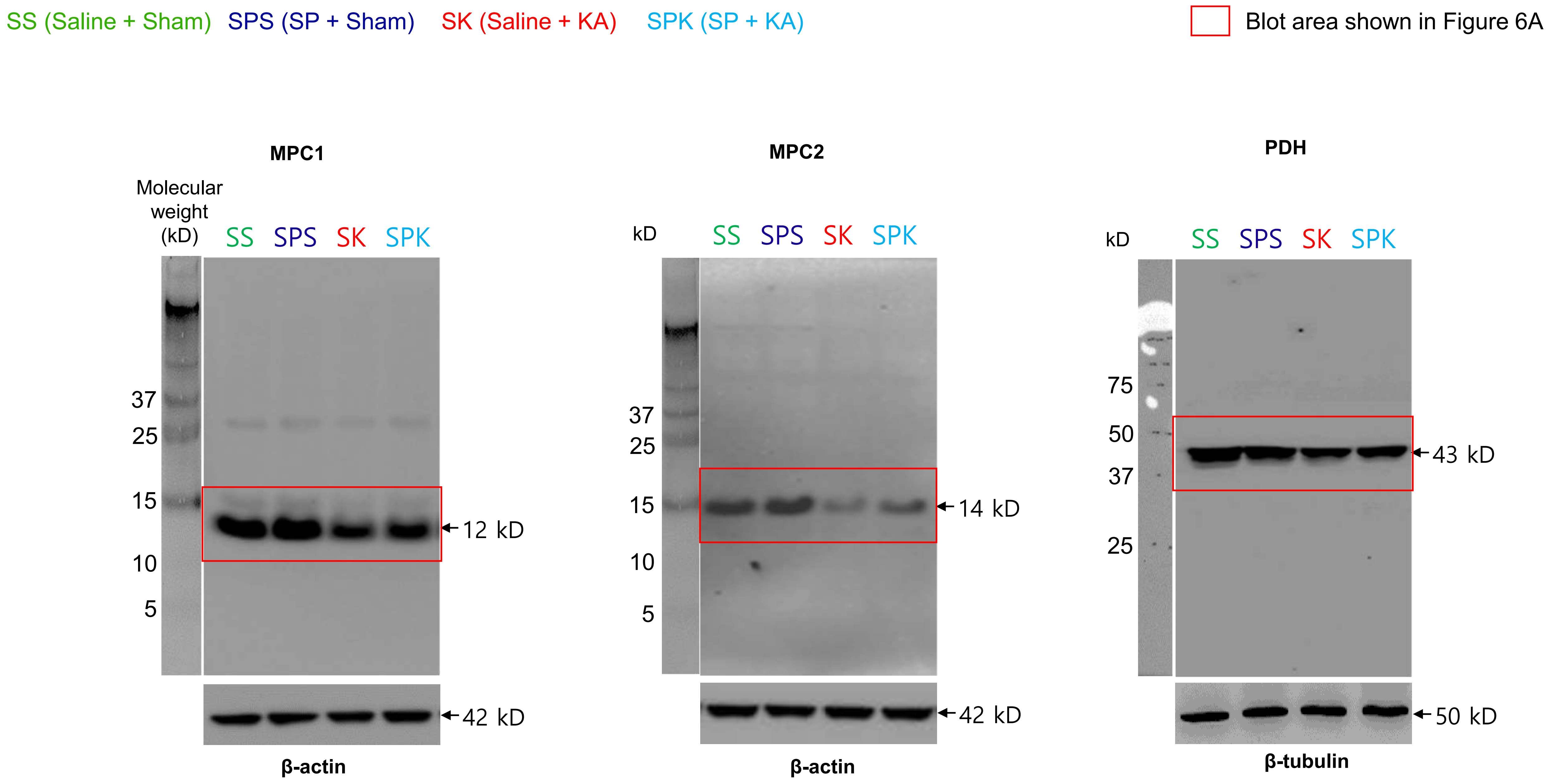
SS (Saline + Sham)
SPS (SP + Sham)
SK (Saline + KA)
SPK (SP + KA)
Blot area shown in Figure 6A
MPC1
Molecular weight (kD)
SS SPS SK SPK
37
25
15
10
5
12 kD
42 kD
β-actin
MPC2
kD
SS SPS SK SPK
37
25
15
10
5
14 kD
42 kD
β-actin
PDH
kD
SS SPS SK SPK
75
50
37
25
43 kD
50 kD
β-tubulin

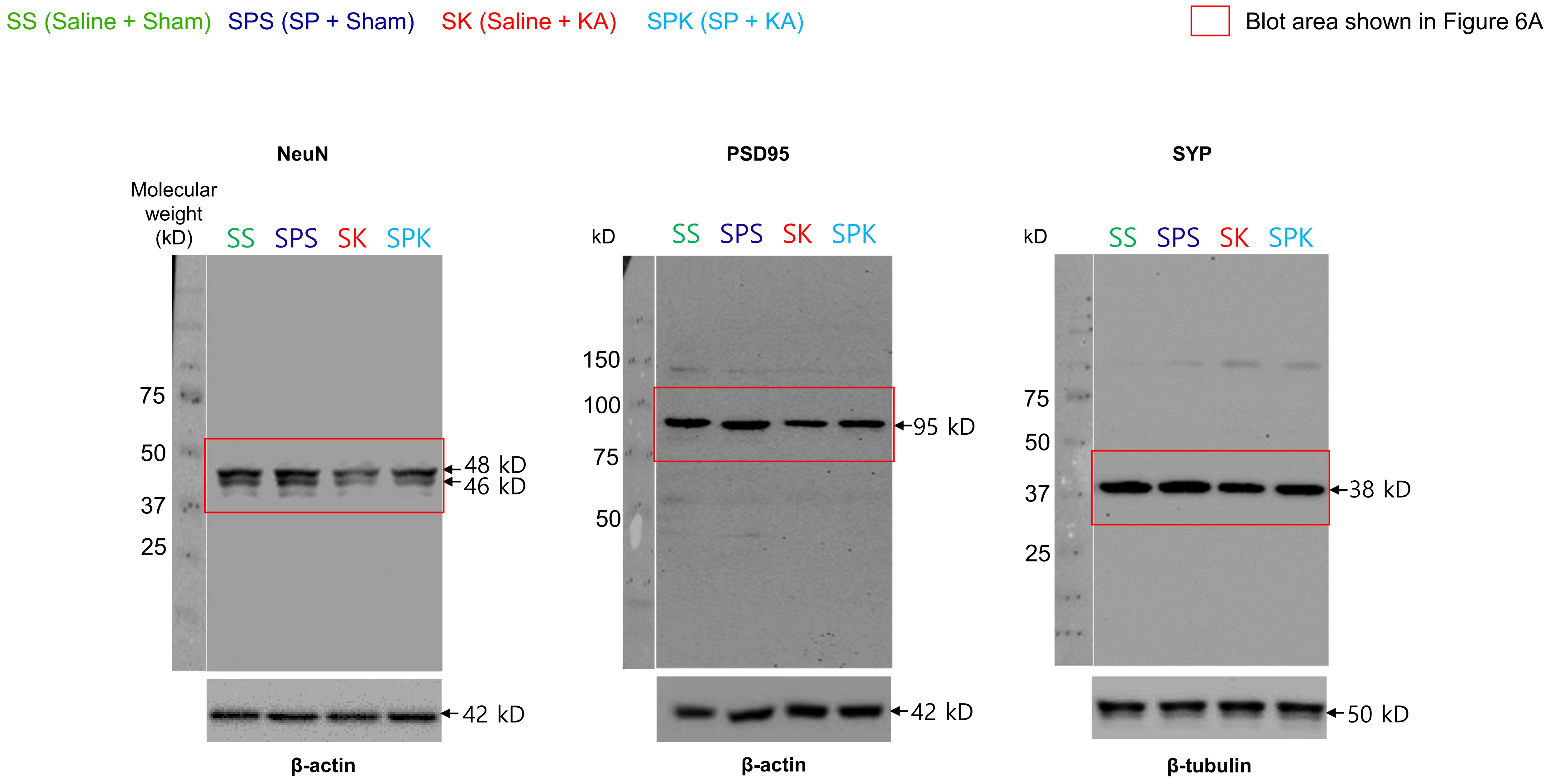

SS (Saline + Sham)
SPS (SP + Sham)
SK (Saline + KA)
SPK (SP + KA)
Blot area shown in Figure 6A
NeuN
Molecular weight (kD)
SS
SPS
SK
SPK
75
50
37
25
48 kD
46 kD
42 kD
β-actin
PSD95
kD
150
100
75
50
95 kD
42 kD
β-actin
SYP
kD
75
50
37
25
38 kD
50 kD
β-tubulin

SS (Saline + Sham) SPS (SP + Sham) SK (Saline + KA) SPK (SP + KA) Blot area shown in Figure 6A

Caspase 3 and cleaved caspase 3

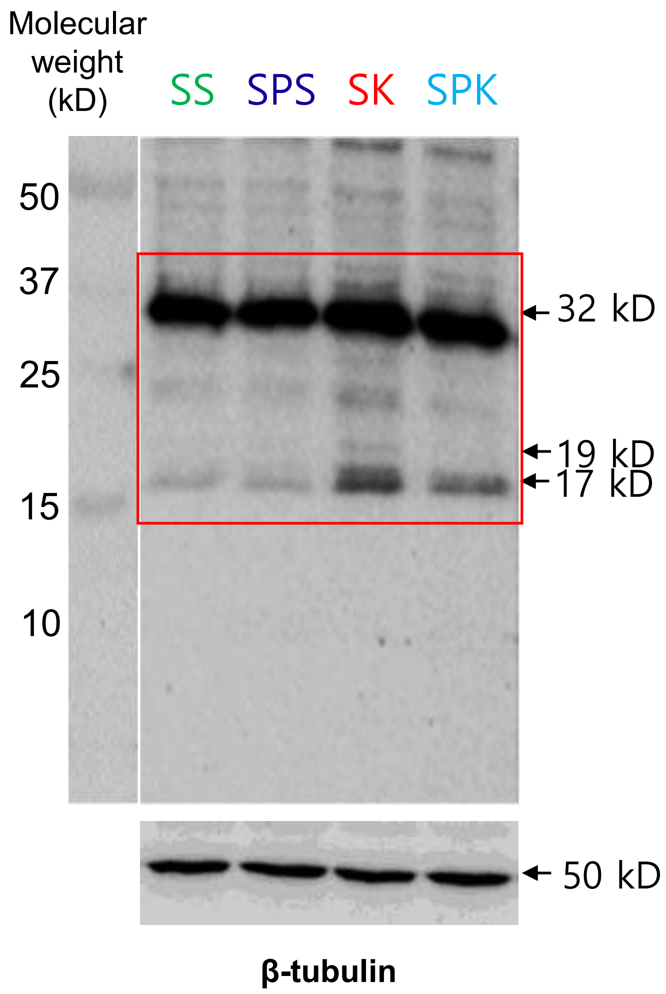

PARP 1 and cleaved PARP 1

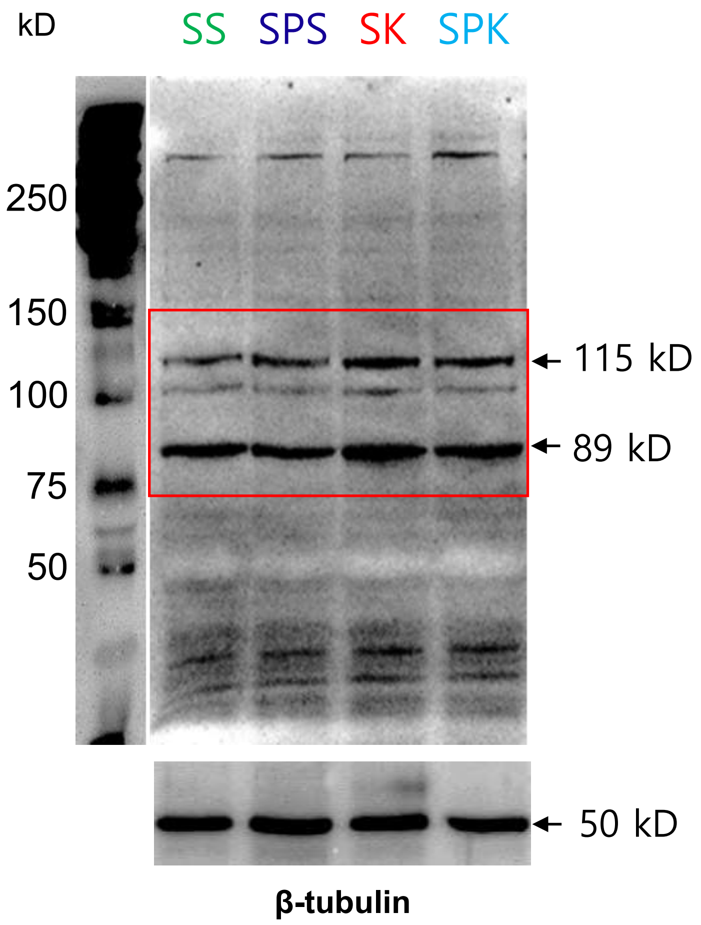

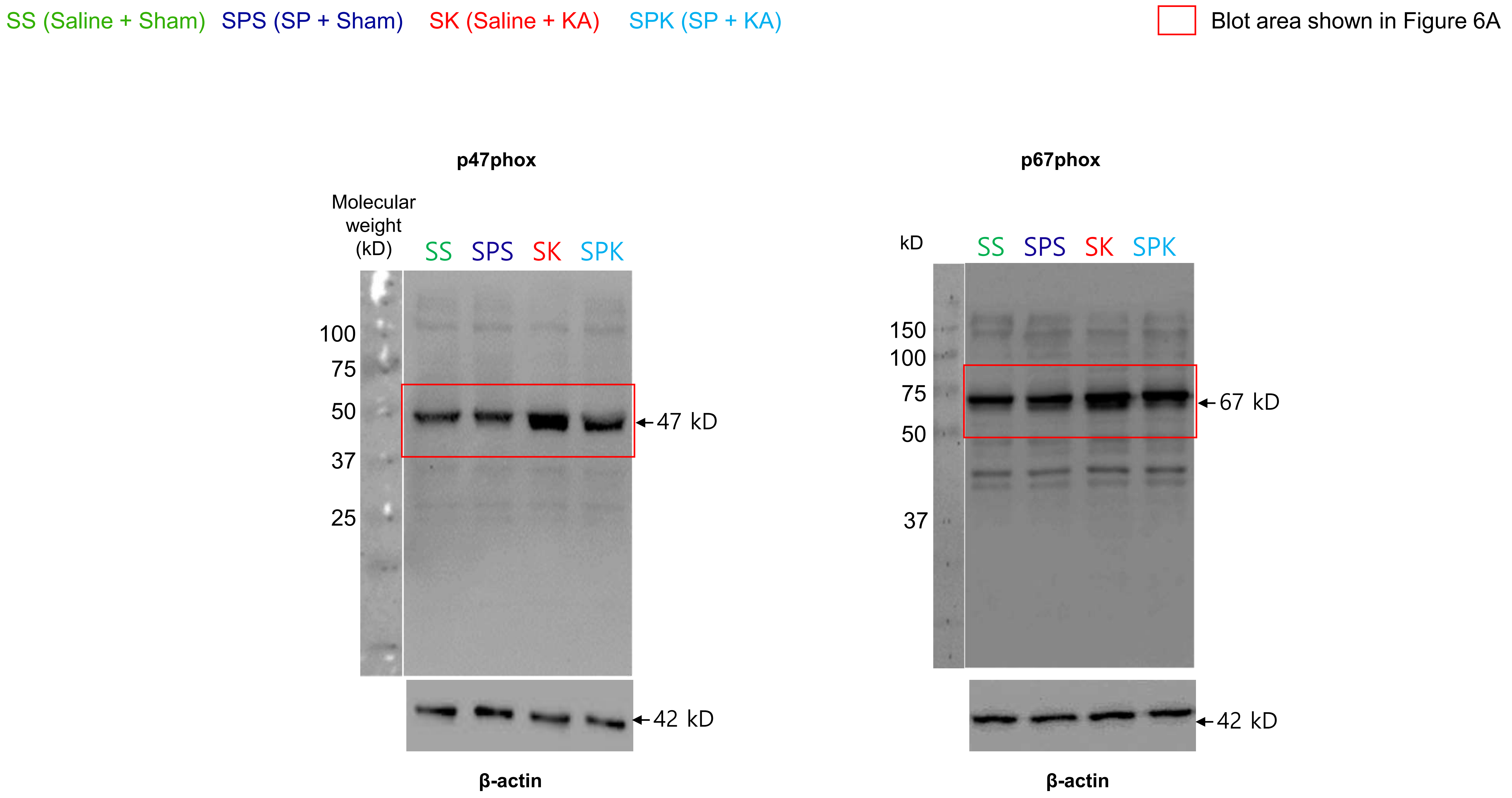

SS (Saline + Sham)
SPS (SP + Sham)
SK (Saline + KA)
SPK (SP + KA)
Blot area shown in Figure 6A
p47phox
Molecular weight (kD)
SS
SPS
SK
SPK
100
75
50
37
25
47 kD
42 kD
β-actin
p67phox
kD
SS
SPS
SK
SPK
150
100
75
50
37
67 kD
42 kD
β-actin

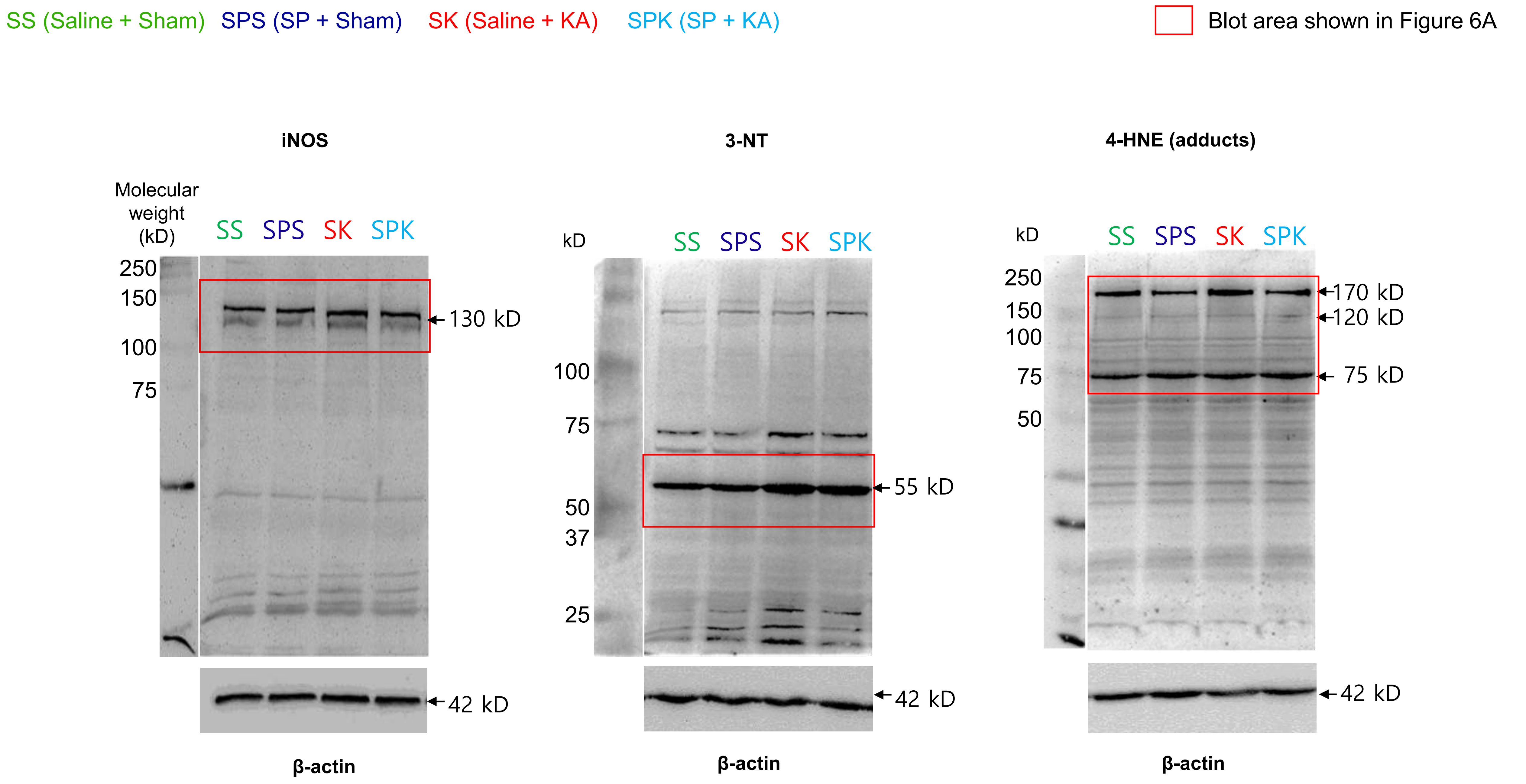
SS (Saline + Sham)
SPS (SP + Sham)
SK (Saline + KA)
SPK (SP + KA)
Blot area shown in Figure 6A
iNOS
Molecular weight (kD)
SS SPS SK SPK
250
150
100
75
130 kD
42 kD
β-actin
3-NT
kD
SS SPS SK SPK
100
75
50
37
25
55 kD
42 kD
β-actin
4-HNE (adducts)
kD
SS SPS SK SPK
250
150
100
75
50
170 kD
120 kD
75 kD
42 kD
β-actin

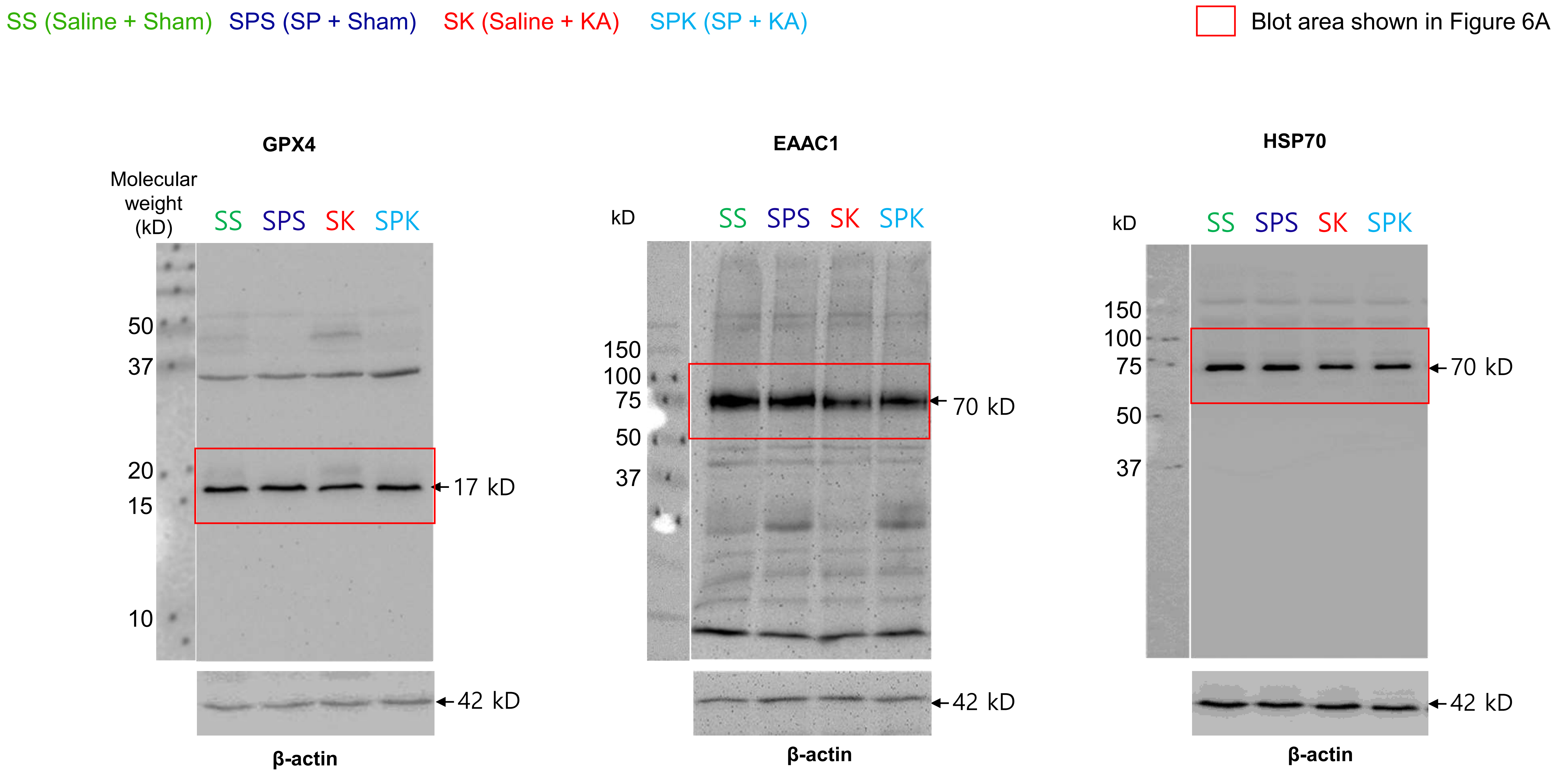
SS (Saline + Sham) SPS (SP + Sham) SK (Saline + KA) SPK (SP + KA)
Blot area shown in Figure 6A
GPX4
Molecular weight (kD)
SS SPS SK SPK
50
37
20
15
10
17 kD
42 kD
β-actin
EAAC1
kD
SS SPS SK SPK
150
100
75
50
37
70 kD
42 kD
β-actin
HSP70
kD
SS SPS SK SPK
150
100
75
50
37
70 kD
42 kD
β-actin

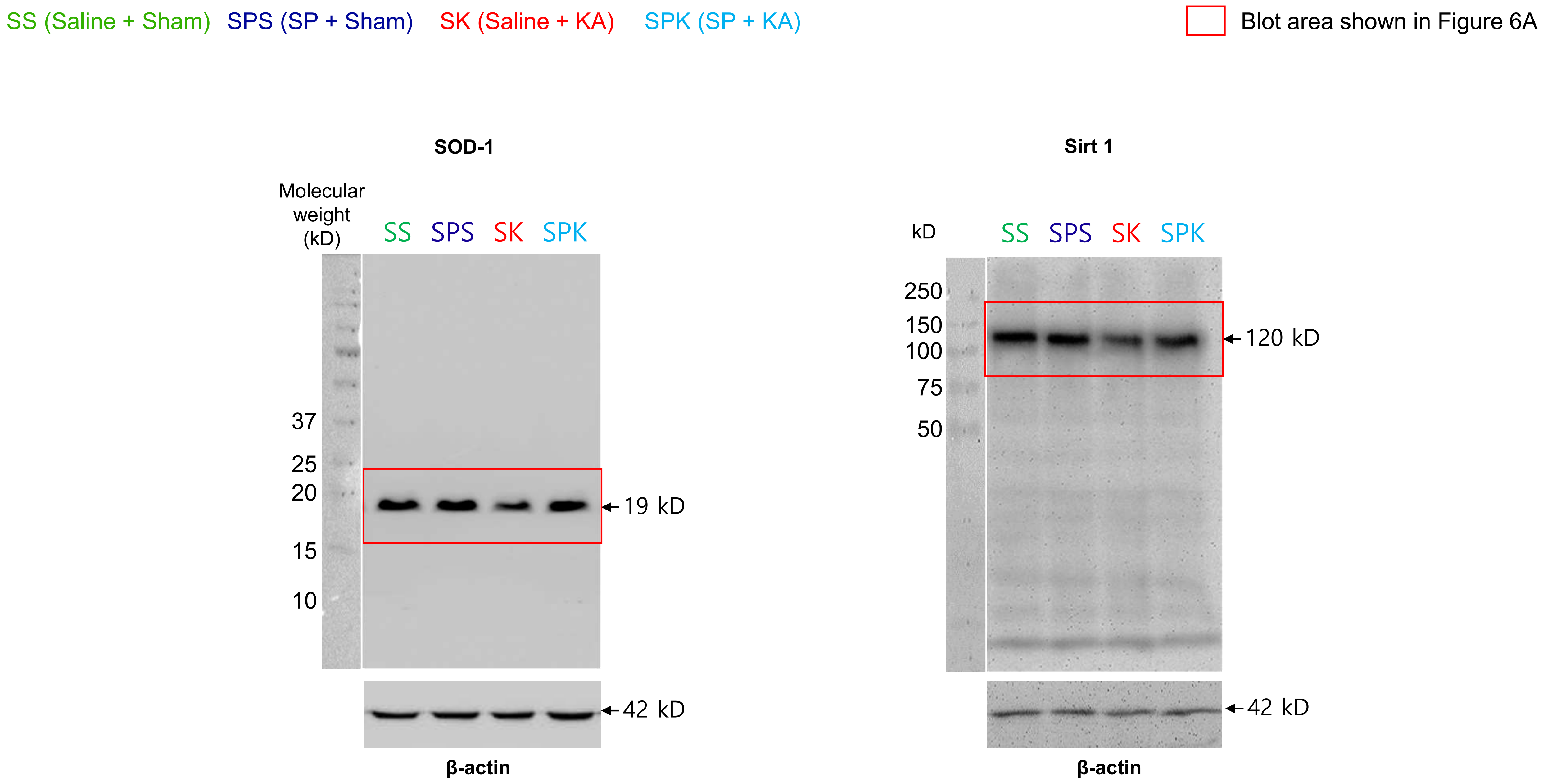
SS (Saline + Sham)
SPS (SP + Sham)
SK (Saline + KA)
SPK (SP + KA)
Blot area shown in Figure 6A
SOD-1
Molecular weight (kD)
SS
SPS
SK
SPK
37
25
20
15
10
19 kD
42 kD
β-actin
Sirt 1
kD
SS
SPS
SK
SPK
250
150
100
75
50
120 kD
42 kD
β-actin
