# Supplementary material for: Continuous Pyruvate Supplementation Enhances Neuroprotective Resilience Against Kainate-Induced Status Epilepticus Through Metabolic Preconditioning
Source: Biomolecules. 2026 May 29;16(6):805. doi: 10.3390/biom16060805 (PMC13297230; doi:10.3390/biom16060805)
Supplement: Supplementary file 1 [file biomolecules-16-00805-s001.zip › Table S1.pdf]

Table S1. Concentrations and CRLB values of metabolic neurochemicals measured using *in vivo* ^{1}H-MRS in the hippocampus of the normal mice fed either saline solution or 3% sodium pyruvate (SP)-supplemented water

	Saline-fed (n = 11)		SP-fed (n = 7)			
	Concentration (mM)	CRLB (%SD)	Concentration (mM)	CRLB (%SD)	% vs. Saline-fed	P value[#]
PCr	7.325 ± 1.708[*]	6.909 ± 2.119	9.389 ± 1.613	7.429 ± 2.879	128.2	0.021
GABA	2.750 ± 0.356	14.000 ± 2.757	3.261 ± 0.328	13.714 ± 1.604	118.6	0.008
Gln	4.691 ± 0.424	8.727 ± 1.191	5.362 ± 0.922	8.571 ± 1.618	114.3	0.051
Glu	11.192 ± 1.025	3.818 ± 0.874	12.860 ± 2.030	3.571 ± 0.535	114.9	0.034
GSH	2.999 ± 0.375	7.364 ± 1.804	3.635 ± 0.327	7.288 ± 1.496	121.2	0.002
MI	6.435 ± 0.676	4.909 ± 1.514	7.742 ± 1.467	5.000 ± 1.291	120.3	0.020
NAA	7.583 ± 1.213	3.727 ± 0.647	9.401 ± 1.539	3.714 ± 0.756	124.0	0.013
TRN	15.328 ± 1.357	2.545 ± 0.522	18.188 ± 2.896	2.714 ± 0.488	118.7	0.011
tCho	1.980 ± 0.119	4.000 ± 0.894	1.917 ± 0.244	4.429 ± 0.976	96.8	0.468
tNAA	8.808 ± 1.050	3.727 ± 0.786	10.177 ± 1.755	3.572 ± 0.787	115.5	0.053
tCr	11.454 ± 1.238	2.455 ± 0.688	13.347 ± 1.491	2.714 ± 0.488	116.5	0.010
Glx	15.883 ± 1.317	3.636 ± 0.674	18.094 ± 2.211	3.714 ± 0.488	113.9	0.017

[*] Data are mean values ± SD. [#] P values were calculated using the unpaired t-test.

^{1}H-MRS, proton magnetic resonance spectroscopy; n, animal number; CRLB, Cramér-Rao Lower Bound; SP, sodium pyruvate; PCr, phosphocreatine; GABA, γ-aminobutyric acid; Gln, glutamine; Glu, glutamate; GSH, glutathione; MI, myo-inositol; NAA, N-acetylaspartate; TRN, taurine; tCho (GPC + PCh), total choline (glycerophosphocholine + phosphorylcholine); tNAA (NAA + NAAG), total NAA (NAA + N-acetylaspartylglutamate); tCr (Cr + PCr), total creatine (creatine + phosphocreatine); Glx (Gln + Glu), glutamate complex (glutamine + glutamate).
